# Fatimanols Y and Z: two *neo*-clerodane diterpenoids from *Teucrium yemense*†

**DOI:** 10.1039/d3ra06083g

**Published:** 2023-10-20

**Authors:** Ahmed Elbermawi, Fazila Zulfiqar, Ikhlas A. Khan, Zulfiqar Ali

**Affiliations:** a Department of Pharmacognosy, Faculty of Pharmacy, Mansoura University Mansoura 35516 Egypt asbeder@mans.edu.eg +20 10-0481-1533; b National Center for Natural Products Research, School of Pharmacy, University of Mississippi, University MS 38677 USA

## Abstract

*Teucrium yemense* (Defl.), a medicinal plant, grows in Yemen and Saudi Arabia and is also referred to as Reehal Fatima. The plant has a long history of use in these regions for the treatment of diabetes, rheumatism, and renal conditions. Phytochemical investigation of the aerial parts of *T. yemense* yielded two previously undescribed *neo*-clerodane diterpenoids, namely fatimanols Y and Z (1 and 2) along with the known teulepicephin (3), 8-acetylharpagide (4) and teucardosid (5). Structure elucidation was accomplished from their 1D and 2D NMR, ECD, and MS characteristics as well as by comparing them to related reported compounds. The new molecules expand understanding of secondary metabolites of this genus. Compounds 1–5 did not show antimicrobial activity against various bacterial and fungal strains.

## Abstract 2

*Teucrium yemense* (Defl.), a medicinal plant, grows in Yemen and Saudi Arabia and is also referred to as Reehal Fatima. The plant has a long history of use in these regions for the treatment of diabetes, rheumatism, and renal conditions.

## Graphical abstract

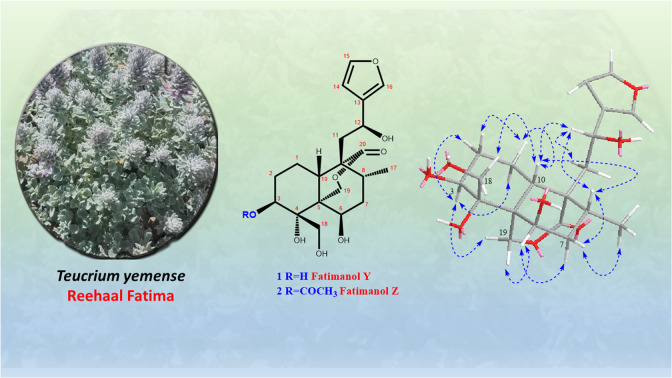

## 1. Introduction

*Teucrium* L. (Lamiaceae), commonly known as germanders, is a cosmopolitan genus of about 300 species mainly distributed in South and Central America, Southern Asia, and the Middle East but predominantly prevalent in the Mediterranean basin. Plants in this genus are generally perennial, herbs or shrubs, and the corollas are mostly white to cream-colored with characteristic reduced upper lips.^1,2^*Teucrium* species have been used traditionally as diuretic, diaphoretic, antipyretic, and antiseptic agents for centuries in many parts of the world.^3^ Several biological activities such as anthelmintic, insecticide, antiulcer, antispasmodic, analgesic, antioxidant, anti-inflammatory, antifeedant, and antimicrobial have been related to *Teucrium*.^4–7^ In Egypt, *Teucrium* is used as an appetizer, expectorant, and hypoglycemic.^8^ About 300 compounds including flavonoids, terpenoids, iridoids, steroids, phenylethanoids and mainly diterpenoids have been reported from different species of *Teucrium*. The *Teucrium* genus is a rich source of diterpenoids, particularly *neo*-clerodanes which are used as chemotaxonomic markers for *Teucrium* species. More than 220 diterpenes have been described so far from *Teucrium*.^9^

*Teucrium yemense* (Defl.), commonly known as Reehal Fatima, is a therapeutic plant that is frequently grown in Yemen and Saudi Arabia. The plant has a long history of use in these areas for the treatment of diabetes, rheumatism, and renal ailments.^10–12^ Over thirty *neo*-clerodane diterpene derivatives from this species have been identified and four of them showed potential antidiabetic activity.^2,13,14^ Based on the aforementioned facts, the aerial parts of *T. yemense* were selected to explore further chemical investigation.

## 2. Result and discussion

**Fig. 1:**
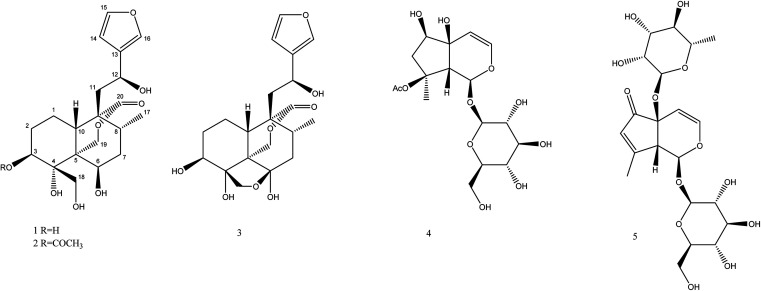
Structures of compounds 1–5.

Using a combination of chromatographic techniques, five compounds (1–5) (Fig. 1) were isolated from the methanolic extract of the aerial parts of *T. yemense*. Compounds 1 and 2 (fatimanols Y and Z) were previously undescribed and were identified as (12*S*)-15,16-epoxy-3β,4α,6β,12-tetrahydroxy-18-hydroxy-*neo*-cleroda-13(16),14-dien-20,19-olide and (12*S*)-15,16-epoxy-3β-acetyl-4α,6β,12-tetrahydroxy-18-hydroxy-*neo*-cleroda-13(16),14-dien-20,19-olide, respectively, based on 1D and 2D NMR spectroscopic and mass spectral data.

**Fig. 2:**
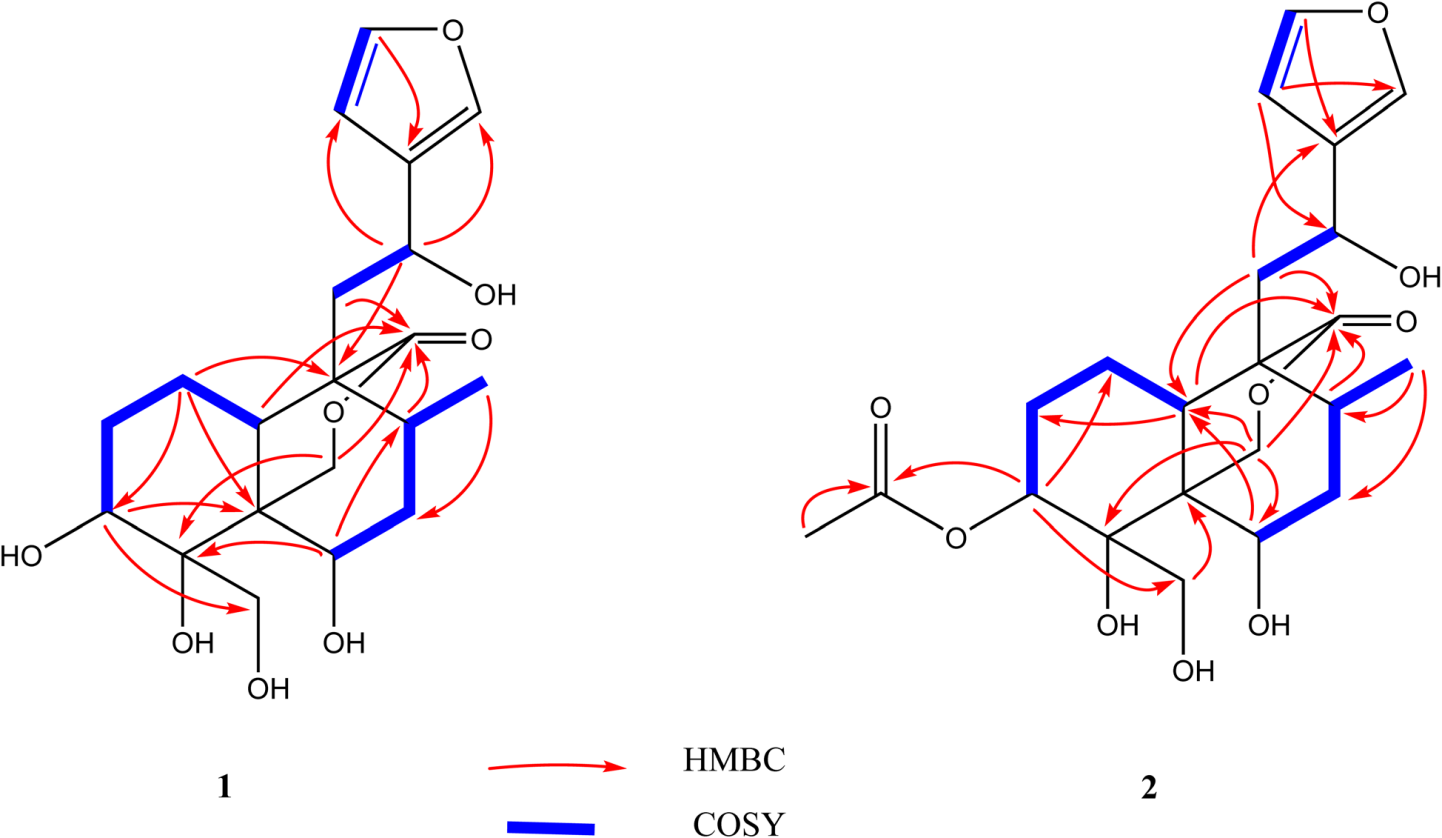
Key HMBC and COSY correlations of compounds 1 and 2.

Compound 1 was obtained as colorless gum with a molecular formula C_20_H_28_O_8_ deduced on the basis an [M–H]^−^ ion peak in the HRESIMS at *m*/*z* 395.1702 (calcd *m*/*z* 395.1711) and the number of carbon resonances in the DEPTQ-135 spectrum. The NMR data showed resonances, typical for a furanyl moiety [*δ*_H/C_ 6.49 (d, 1.9 Hz)/109.7 (CH-14), 7.47 (t, 1.7 Hz)/144.6 (CH-15), and 7.49 (d, 1.6 Hz)/139.9 (CH-16) and *δ*_C_ 132.1 (C-13)], a methyl [*δ*_H/C_ 0.80 (d, 6.8 Hz)/16.8 (CH_3_-17)], three oxy-methines [*δ*_H/C_ 4.06/72.9 (CH-3), 4.15/69.4 (CH-6), and 4.86/63.3 (CH-12)], two isolated oxy-methylenes [*δ*_H/C_ 3.96, 4.05/59.9 (CH_2_-18), and 3.97, 4.74/74.5 (CH_2_-19)], an ester carbonyl [*δ*_C_ 175.8 (C-20)], and an oxy-nonprotonated carbon [*δ*_C_ 77.3 (C-4)]. Besides, resonances in the aliphatic region for four methylenes, two methines, and two quaternary carbons were also observed. Based on the NMR data (Table 1), a *neo*-clerodane diterpenoid skeleton was assumed for 1. The presence of two isolated oxy-methylenes (CH_2_-18 and CH_2_-19)] and a carbonyl (C-20) indicated that three methyl groups of *neo*-clerodane were oxidized. The locations of oxygenated methines were supported by the HMBC correlations of H-12 (*δ*_H_ 4.86) with C-14 (*δ*_C_ 109.7), C-16 (*δ*_C_ 139.9), and C-9 (*δ*_C_ 51.3); H-3 (*δ*_H_ 4.06) with C-4 (*δ*_C_ 77.3), C-5 (*δ*_C_ 46.1), and C-18 (*δ*_C_ 59.9); and H-6 (*δ*_H_ 4.15) with C-4 (*δ*_C_ 77.3), C-5 (*δ*_C_ 46.1), and C-8 (*δ*_C_ 30.2) (Fig. 2). The C-18 and C-19 oxy-methylenes as well as non-protonated oxycarbon C-4 were confirmed by the HMBC correlations of H_2_-18 with C-3, C-4, and C-5 and H_2_-19 with C-4 and C-5. The HMBC correlations of H_2_-19 (oxy-methylene), H_2_-11, and H-10 with carbonyl (*δ*_C_ 175.8) supported five-membered lactone (C-5–C-19–O–C-20–C-9–C-10).

**Table 1:** ^1^H NMR & ^13^C NMR data (in CD_3_OD) of compounds 1 and 2

	Compound 1		Compound 2	
Position	*δ*_C_	*δ*_H_ a mult. (*J* in Hz)	*δ*_C_	*δ*_H_ a mult. (*J* in Hz)
1	25.3	1.12 m	24.9	1.19 m
		2.44 dq (13.1, 3.8)		2.48 dq (13.5, 2.5)
2	29.9	1.46 qd (13.1, 3.9)	27.6	1.71
		1.82		1.87
3	72.9	4.06 dd (12.6, 4.6)	75.9	5.28 dd (12.7, 4.8)
4	77.3		76.3	
5	46.1		46.7	
6	69.4	4.15 br. t (2.9)	69.2	4.20 br. t (2.8)
7	37.5	1.63 ddd (14.9, 12.8, 2.5)	37.6	1.63
		1.83		1.82
8	30.2	2.59 m	30.2	2.64 m
9	51.3		51.2	
10	35.8	2.93 dd (13.1, 4.5)	35.8	3.04 dd (13.0, 4.4)
11	37.2	2.03 dd (15.8, 7.3)	37.2	2.06
		2.37 dd (15.8, 3.4)		2.38 dd (15.8, 3.3)
12	63.3	4.86	63.3	4.86
13	132.1		132.1	
14	109.7	6.49 d (1.9)	109.7	6.50 dd (2.0, 0.9)
15	144.6	7.47 br. t (1.7)	144.6	7.48 br. t (1.7)
16	139.9	7.49 br. s	139.9	7.50 d (1.6)
17	16.8	0.80 d (6.8)	16.8	0.82 d (6.8)
18	59.9	3.96 d (11.5)	61.4	3.91 d (11.7)
		4.05 d (11.5)		4.10 d (11.7)
19	74.5	3.97 d (13.8)	74.3	4.03 d (13.9)
		4.74 d (13.8)		4.77 d (13.9)
20	175.8		175.4	
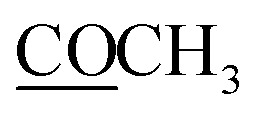			172.5	
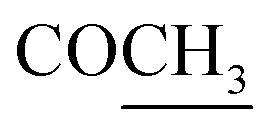			21.2	2.11 s

a Multiplicity is not clear for some signals due to overlapping.

**Fig. 3:**
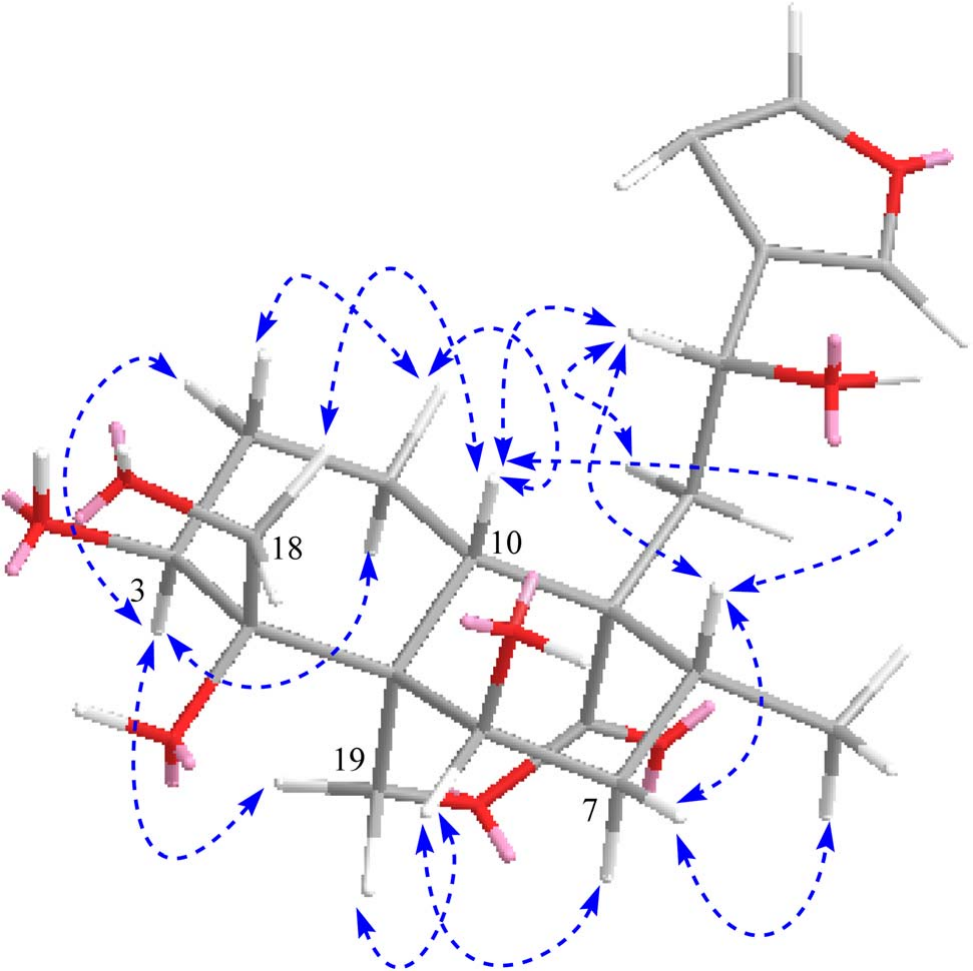
Key NOESY correlations of both compounds 1 and 2.

The absolute configuration at C-12 was determined to be *S* due to the negative cotton effect at 242–260 nm in the experimental ECD spectrum of 1 (Fig. S34†) as it had been reported so by Aydoğan *et al.* based on the experimental and calculated ECD data of teusandrin H.^15^ The relative stereochemistry of the other chiral points was determined based on the NOESY correlations (Fig. 3) and characteristic coupling constant values. The characteristic larger coupling constant (13.1 Hz) exhibited by biogenetically β-faced H-10 with H-1_ax_ supported its axial orientation 10*S* configuration. Similarly, the larger coupling constant value of H-3 (12.6 Hz) with H-2_ax_ and smaller coupling constant value of H-6 (br. t, 2.9 Hz) with H-7_ax/eq_ supported the axial and equatorial orientations of H-3 and H-6, respectively, which ultimately revealed equatorially oriented OH-3 and axially orientated OH-6 with 3*S* and 6*R* configurations. The NOESY correlations of H-10_ax_ with H-8_ax_/H-12/H-18 revealed their co-faced orientations with 8*R*, 12*S*, 4*R* configurations. Similarly, the NOESY correlation of H-3_ax_ with H-1_ax_/H-19, and H-6 with H-19/H-7_ax_ confirmed their co-faced assimilation on the other side of the plane and eventually supported 5*R* and 9*R* configurations (Fig. 3). The NMR data of 1 were comparable to those of teuluteumin A except for the missing resonances of methoxy group.^16^ Ultimately, compound 1 was elucidated as (3*S*,4*R*,5*R*,6*R*,8*R*,9*R*,10*S*,12*S*)-15,16-epoxy-3,4,6,12-tetrahydroxy-18-hydroxy-*neo*-cleroda-13(16),14-dien-20,19-olide and named fatimanol Y.

Compound 2 exhibited an [M–H]^−^ ion peak in the HRESIMS at *m*/*z* 437.1816 (calcd for C_22_H_29_O_9_, 437.1817) corresponding to the molecular formula of C_22_H_30_O_9_. The NMR data of 2 was comparable to 1 except for the additional resonances [*δ*_H/C_ 2.11/21.2 (CH_3_) and *δ*_C_ 172.5 (carbonyl)] of an acetyl group in 2. The acetyl group was located as an acetoxy group at C-3 based on the HMBC correlations of H-3 (*δ*_H_ 5.28) and methyl group (*δ*_H_ 2.11) with carbonyl (*δ*_C_ 172.5). The complete assignment of ^1^H and ^13^C NMR resonances was accomplished by HSQC, COSY, and HMBC spectroscopic data. Based on the ECD spectrum (Fig. S35†), the NOESY correlations (Fig. 3), and characteristic coupling constant values, the stereogenic centers of 2 were defined similarly as described for compound 1. Thus compound 2 was elucidated as (3*S*,4*R*,5*R*,6*R*,8*R*,9*R*,10*S*,12*S*)-15,16-epoxy-3 acetyl-4,6,12-tetrahydroxy-18-hydroxy-*neo*-cleroda-13(16),14-dien-20,19-olide and named fatimanol Z.

Based on the NMR and HRESIMS data analysis as well as by comparing with the literature data, the known compounds were identified as teulepicephin (3),^2^ 8-acetylharpagide (4),^17^ teucardosid (5).^18^

All isolates were screened for *in vitro* antimicrobial activities. None of the isolated metabolites showed significant antimicrobial activity (up to 20 μg mL^−1^) against *Candida albicans*, *Aspergillus fumigatus*, *Cryptococcus neoformans*, methicillin-resistant *Staphylococcus aureus* (MRS), *Escherichia coli*, *Pseudomonas aeruginosa*, *Klebsiella pneumonia* and *Enterococcus faecium* (VRE).

## 3. Material and methods

### 3.1. General experimental procedure

Optical rotations were measured in MeOH using AUTOPOL II Automatic Polarimeter (Rudolph, Hackettstown, NJ, USA). ECD spectra were collected using Olis DSM 20 CD digital spectropolarimeter (Bogart, GA, USA). IR spectra were determined on an Agilent Technologies Cary 630 FTIR. UV spectra were measured on a Thermo Scientific Evolution 201 UV-visible spectrophotometer. NMR experiments were carried out on a Bruker Avance III 400 MHz spectrometer using CD_3_OD as a solvent and methanol residue signals were used as the internal standard. An Agilent Technologies 6200 series mass spectrometer was employed to acquire mass data. Column chromatography (CC) was performed over flash silica gel (SiliaFlashV^®^P60, SiliCycle Inc., USA). Analytical TLC was carried out on silica gel F_254_ aluminum sheet (20 cm × 20 cm, SiliCycle, Canada) or reversed phase C-18 aluminum sheet (20 cm × 20 cm, Sorbent Tech., USA). The detection of the spots was made possible by visualization under UV-254 nm and by spraying with 1% vanillin in H_2_SO_4_–EtOH (10 : 90), followed by heating. Analytical grade solvents (Fischer chemicals) were used for the isolation and purification procedures.

### 3.2. Plant material

*Teucrium yemense* (Defl.) aerial parts were collected from Abha, Saudi Arabia in March 2013. The plant identity was confirmed by a taxonomist at the College of Pharmacy, King Saud University, Riyadh, Saudi Arabia. The plant material was air-dried in shade at room temperature. A voucher specimen, coded Ty/018, was kept in the Pharmacognosy Department, Faculty of Pharmacy, Mansoura University.

### 3.3. Extraction and isolation

The powdered air-dried aerial parts (280 g) were extracted by maceration with aqueous MeOH (90%) at room temperature. The dried extract (30 g), obtained on the removal of the solvent by rotary evaporator, was subjected to vacuum liquid chromatography (VLC) over reversed phase C-18 silica gel (30 cm × 5 cm), eluted firstly with 100% H_2_O, then H_2_O–MeOH 90 : 10, followed by increasing the MeOH proportions by 10% till 100% MeOH. Fraction eluted with H_2_O–MeOH 90 : 10 & 80 : 20 were mixed (2.7 g) and chromatographed over silica gel column (4 cm × 120 cm), eluted with DCM–MeOH 95 : 5 (4 L) and 90 : 10 (4 L) to purify compounds 3 (5.6 mg), 4 (124 mg), and 5 (80 mg). Fraction eluted with H_2_O–MeOH 70 : 30 (1.9 g) was subjected to repeated column chromatography [silica gel (3 cm × 120 cm), eluted with EtOAc–DCM–MeOH–H_2_O 15 : 8 : 2 : 0.5 (4 L) and [silica gel (2 cm × 120 cm), eluted with DCM–MeOH 9 : 1 (2 L) to obtain compound 1 (29 mg). Fraction eluted with H_2_O–MeOH 60 : 40 (1.3 g) was subjected to repeated column chromatography [silica gel (3 cm × 120 cm), eluted with EtOAc–DCM–MeOH 15 : 8 : 0.5 (3 L) and [silica gel (2 cm × 120 cm), eluted with DCM–MeOH 9 : 1 (2 L) to obtain compound 2 (22 mg).

#### 3.3.1 Compound 1

Colourless gum; [*α*]^25^_d_ – 3.4 (*c* 1.9, MeOH); UV (MeOH) *λ*_max_ (log *ε*) 210 (6.5) nm; IR *υ* 3378, 2935, 1701, 1202, 875 cm^−1^, ^1^H and ^13^C NMR data, see Table 1; HRESIMS *m*/*z* 395.1702 [M–H]^−^ (calcd for C_20_H_27_O_8_, 395.1711).

#### 3.3.2 Compound 2

Colourless gum; [*α*]^25^_d_ – 2.3 (*c* 3, MeOH); UV (MeOH) *λ*_max_ (log *ε*) 211 (3.7) nm IR *υ* 3403, 2965, 1720, 1239, 1049 cm^−1^, ^1^H and ^13^C NMR data see Table 1; HRESIMS *m*/*z* 437.1816 [M–H]^−^ (calcd for C_22_H_29_O_9_, 437.1817).

### 3.4. *In vitro* antimicrobial activity

The antimicrobial activity of the isolated compounds was evaluated against *Candida albicans*, ATCC 90028, *Aspergillus fumigatus* ATCC 204305, *Cryptococcus neoformans* ATCC 90113, methicillin-resistant *Staphylococcus aureus* ATCC 1708 (MRS), *Escherichia coli* ATCC 2452, *Pseudomonas aeruginosa* ATCCBAA-2018, *Klebsiella pneumonia* ATCC 2146 and *Enterococcus faecium* (VRE) ATCC 700221. From the American Type Culture Collection, the strains were purchased (ATCC, Manassas, VA). An altered version of the Clinical and Laboratory Standards Institute (formerly National Committee for Clinical Laboratory Standards) procedures was used for the susceptibility testing.^19^ A final DMSO concentration of 1% was maintained in the assay while serially diluting all samples in 20% DMSO/saline and transferring them in duplicate to 384 well flat-bottom microplates. Following the McFarland standard, inocula were created by adjusting the OD630 of microbe suspensions in incubation broth.^20^ For *C. albicans* RPMI 1640 (2% dextrose/0.03% glutamine/MOPS at pH 6.0) was used, for *C. neoformans*, Sabouraud dextrose was used, while, cation-adjusted Mueller–Hinton pH 7.0 for MRS, VRE, *E. coli*, *K. pneumonia*, and *P. aeruginosa*, and RPMI 1640 broth (2% dextrose, 0.03% glutamine, buffered with 0.165 M MOPS at pH 7.0) for *A. fumigatus* in accordance with the CLSI procedure, to afford recommended inocula as per CLSI protocol. Each assay contained drug controls for bacteria and fungi. MRS, VRE, E. coli, *K. pneumoniae*, *P. aeruginosa*, *C. albicans*, and *A. fumigatus* were incubated at 35 °C for 48 hours, while *C. neoformans* was incubated at 35 °C for 68–72 hours. A Bio-Tek plate reader was used to record the optical density (530 nm) or fluorescence (544ex/590em) of *A. fumigatus*, VRE, and MRS before and after incubation.

## 4. Conclusion

*Teucrium yemense* (Defl.), known as Reehal Fatima, has lately been identified as a potential source for new *neo*-clerodane diterpenoids. Consequently, this study's goal was to further explore the chemistry of this plant. The present investigation revealed two undescribed *neo*-clerodane diterpenoids, namely fatimanol Y and fatimanol Z, together with the known teulepicephin, 8-acetylharpagide and teucardosid from the aerial parts of *T. yemense*.

## Author contributions

Ahmed Elbermawi: conceptualization, investigation, methodology, writing – original draft. Fazila Zulfiqar: investigation, validation. Ikhlas A. Khan: resources, supervision. Zulfiqar Ali: supervision, review & editing.

## Conflicts of interest

The authors declare no conflicts of interest.

## Supplementary Material

RA-013-D3RA06083G-s001
