# Supplementary material for: Fatimanols Y and Z: two neo-clerodane diterpenoids from Teucrium yemense
Source: RSC Adv. 2023 Oct 20;13(43):30264–8. doi: 10.1039/d3ra06083g (PMC10587887; doi:10.1039/d3ra06083g)

Electronic Supplementary Material (ESI) for RSC Advances.
This journal is © The Royal Society of Chemistry 2023

Fatimanols Y and Z: Two neo-clerodane diterpenoids from *Teucrium yemense*

Ahmed Elbermawi [1,2,*], Fazila Zulfiqar [2], Ikhlas A. Khan [2], Zulfiqar Ali [2]

[1] Department of Pharmacognosy, Faculty of Pharmacy, Mansoura University, Mansoura, 35516 Egypt.
[2] National Center for Natural Products Research, School of Pharmacy, University of Mississippi, University, MS 38677, USA.
[*] Ahmed Elbermawi: asbeder@mans.edu.eg; Tel.: +20 10-0481-1533

Correspondence

Ahmed Elbermawi Ph.D.

Department of Pharmacognosy,
Faculty of Pharmacy, Mansoura University, Mansoura, 35516, Egypt
asbeder@mans.edu.eg

Contents

Fig. S1. HR-ESI$^-$MS [M-H]$^-$ spectrum of compound **1**
Fig. S2. ^{1}H NMR spectrum of compound **1** (CD_3OD, 400 MHz)
Fig. S3. DEPTQ spectrum of compound **1** (CD_3OD, 100 MHz)
Fig. S4. ^{1}H-^{1}H COSY spectrum of compound **1** (CD_3OD, 400 MHz).
Fig. S5. HSQC spectrum of compound **1** (CD_3OD, 400 MHz).
Fig. S6. HMBC spectrum of compound **1** (CD_3OD, 400 MHz).
Fig. S7. NOESY spectrum of compound **1** (CD_3OD, 400 MHz).
Fig. S8. IR spectrum of compound **1**
Fig. S9. HR-ESI$^-$MS [M-H]$^-$ spectrum of compound **2**
Fig. S10. ^{1}H-NMR spectrum of compound **2** (CD_3OD, 400 MHz).
Fig. S11. DEPTQ spectrum of compound **2** (CD_3OD, 100 MHz).
Fig. S12. ^{1}H-^{1}H COSY spectrum of compound **2** (CD_3OD, 400 MHz).
Fig. S13. HSQC spectrum of compound **2** (CD_3OD, 400 MHz).
Fig. S14. HMBC spectrum of compound **2** (CD_3OD, 400 MHz).
Fig. S15. NOESY spectrum of compound **2** (CD_3OD, 400 MHz).
Fig. S16. IR spectrum of compound **2**
Fig. S17. HR-ESI$^-$MS [M-H]$^-$ spectrum of compound **3**
Fig. S18. ^{1}H NMR spectrum of compound **3** (CD_3OD, 400 MHz)
Fig. S19. DEPTQ spectrum of compound **3** (CD_3OD, 100 MHz)
Fig. S20. ^{1}H-^{1}H COSY spectrum of compound **3** (CD_3OD, 400 MHz).
Fig. S21. HSQC spectrum of compound **3** (CD_3OD, 400 MHz).
Fig. S22. HMBC spectrum of compound **3** (CD_3OD, 400 MHz).
Fig. S23. HR-ESI$^-$MS [M-H]$^-$ spectrum of compound **4**
Fig. S24. ^{1}H NMR spectrum of compound **4** (CD_3OD, 400 MHz)
Fig. S25. DEPTQ spectrum of compound **4** (CD_3OD, 100 MHz)
Fig. S26. HR-ESI$^-$MS [M+Na]$^+$ spectrum of compound **5**
Fig. S27. ^{1}H NMR spectrum of compound **5** (CD_3OD, 400 MHz)
Fig. S28. DEPTQ spectrum of compound **5** (CD_3OD, 100 MHz)
Fig. S29. ^{1}H-^{1}H COSY spectrum of compound **5** (CD_3OD, 400 MHz).
Fig. S30. HSQC spectrum of compound **5** (CD_3OD, 400 MHz).
Fig. S31. HMBC spectrum of compound **5** (CD_3OD, 400 MHz).
Fig. S32. Schematic isolation of the compounds (**1-5**) from the aerial parts of *T. yemense*
Fig. S33. HPLC chromatograms of compounds **1** and **2**
Fig. S34. Experimental ECD spectrum of compound **1**
Fig. S35. Experimental ECD spectrum of compound **2**

Fig. S1. HR-ESI⁻MS [M-H]⁻ spectrum of compound **1**

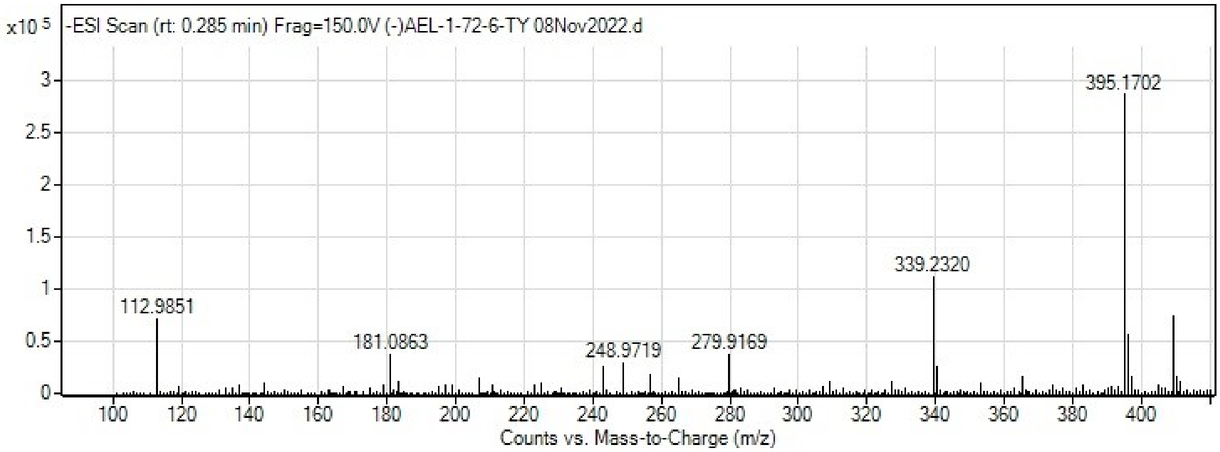

Fig. S2. ^{1}H NMR spectrum of compound **1** (CD_3OD, 400 MHz)

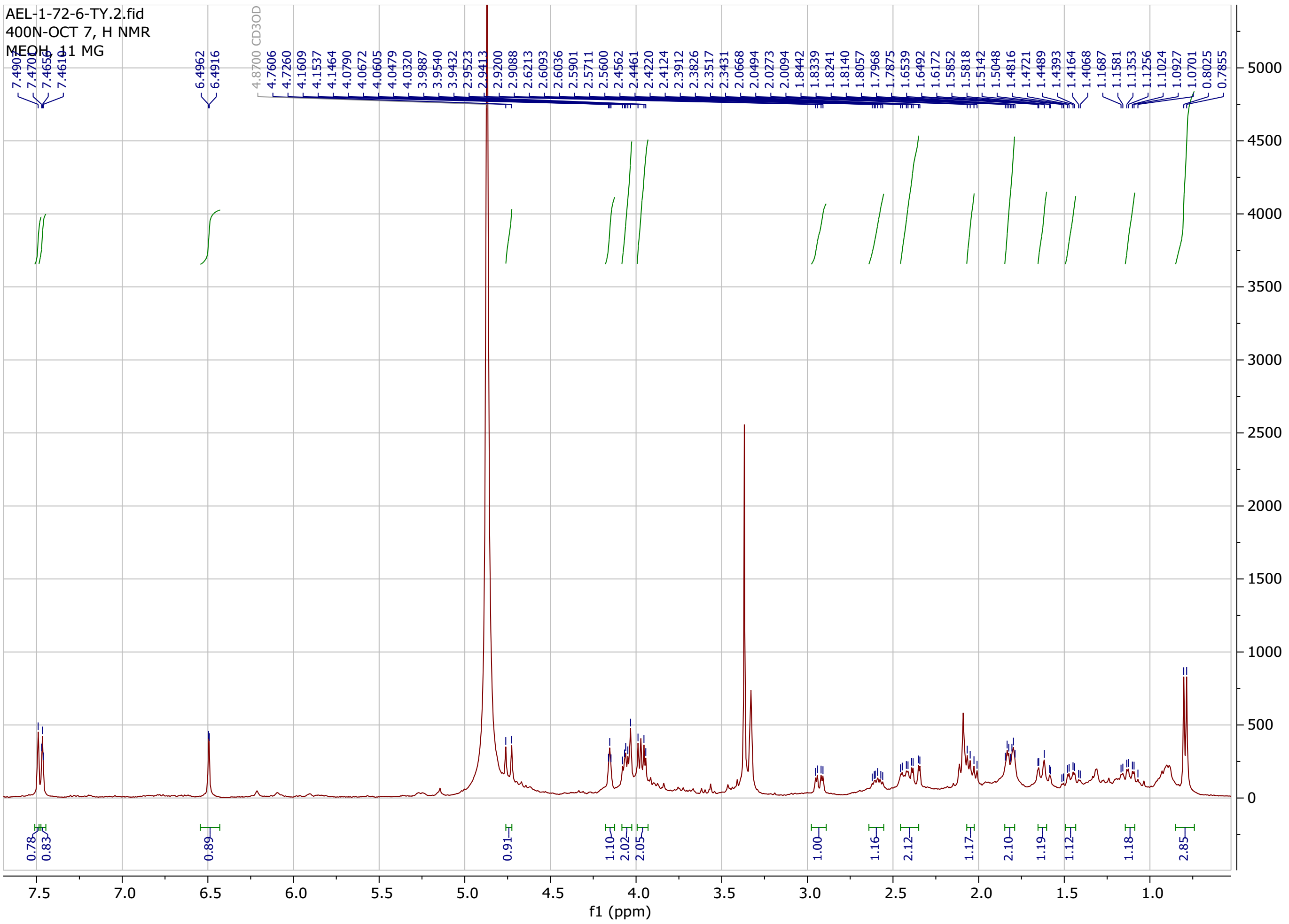

Fig. S3. DEPTQ spectrum of compound **1** (CD_3OD, 100 MHz)

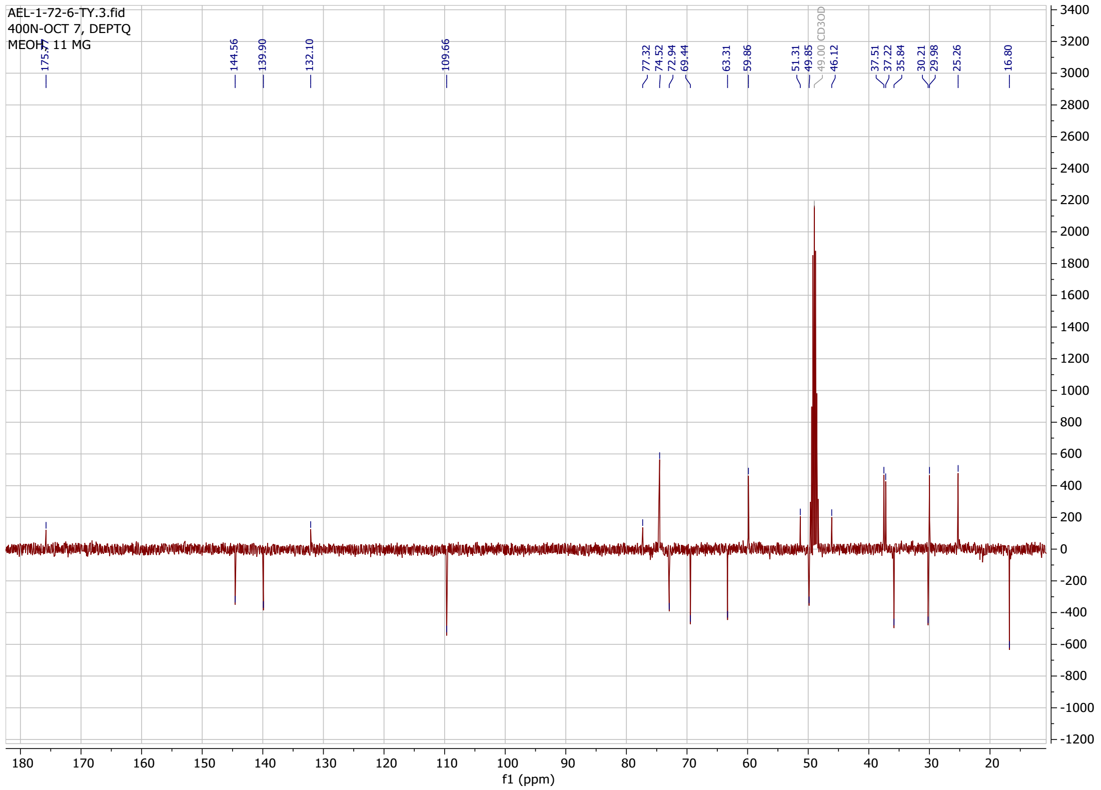

Fig. S4. ^1H-^1H COSY spectrum of compound **1** (CD_3OD, 400 MHz)

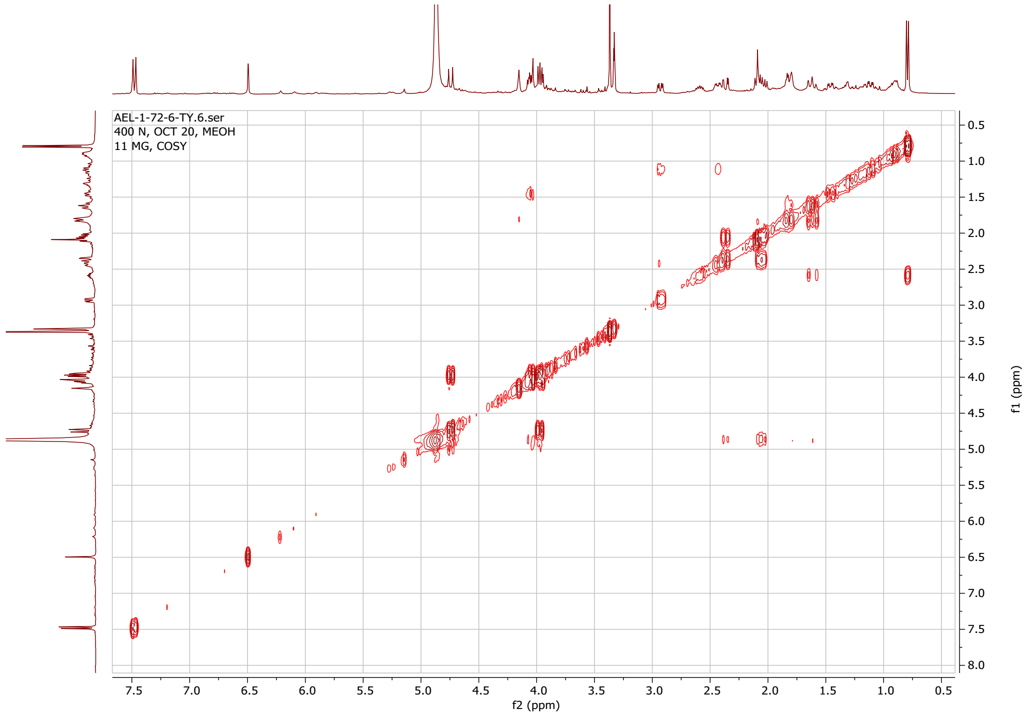

Fig. S5. HSQC spectrum of compound **1** (CD_3OD, 400 MHz)

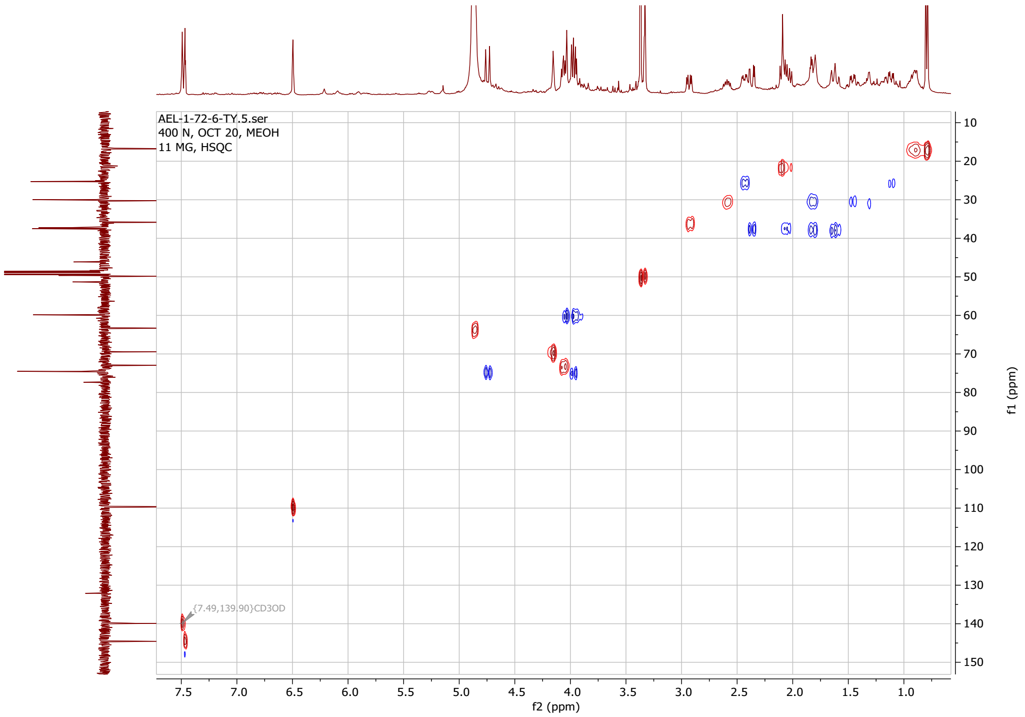

Fig. S6. HMBC spectrum of compound **1** (CD_3OD, 400 MHz)

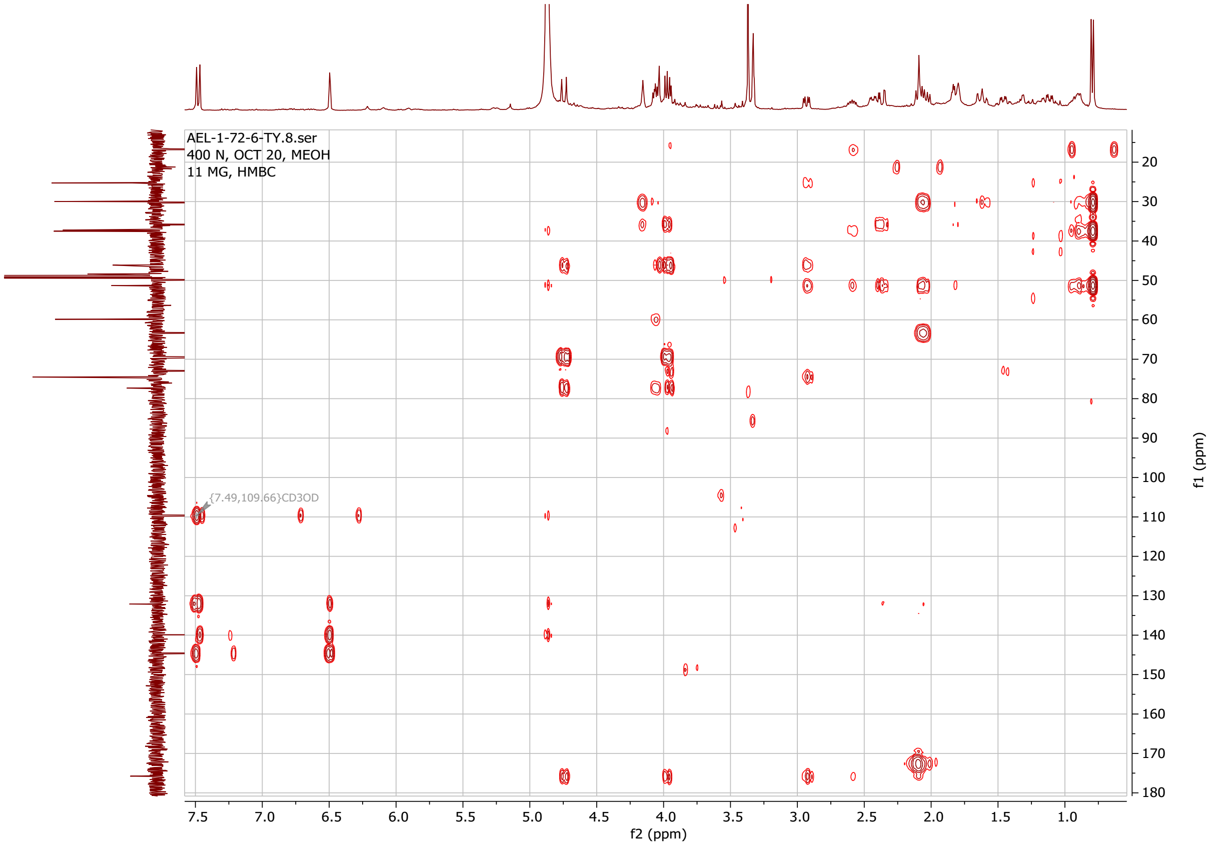

Fig. S7. NOESY spectrum of compound **1** (CD_3OD, 400 MHz)

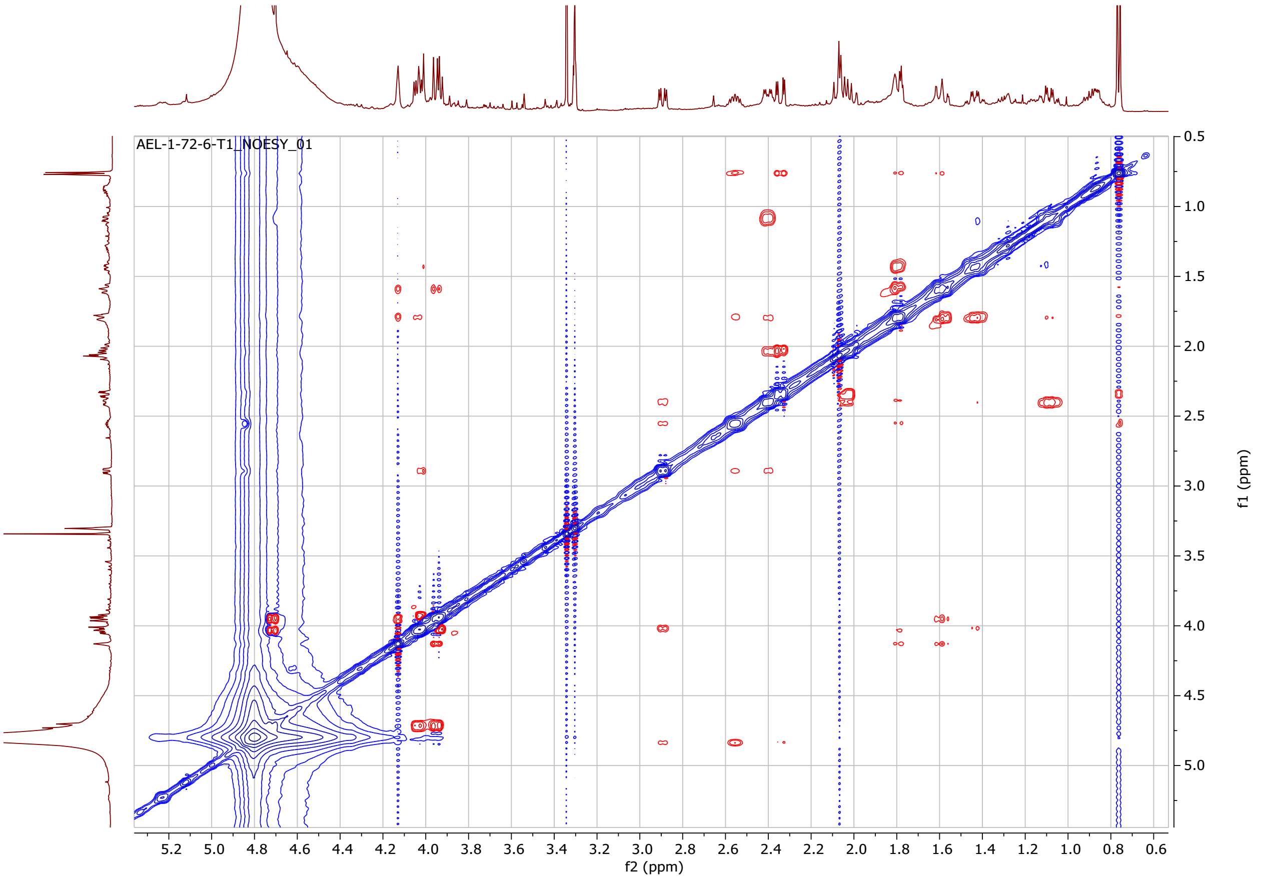

Fig. S8. IR spectrum of compound **1**

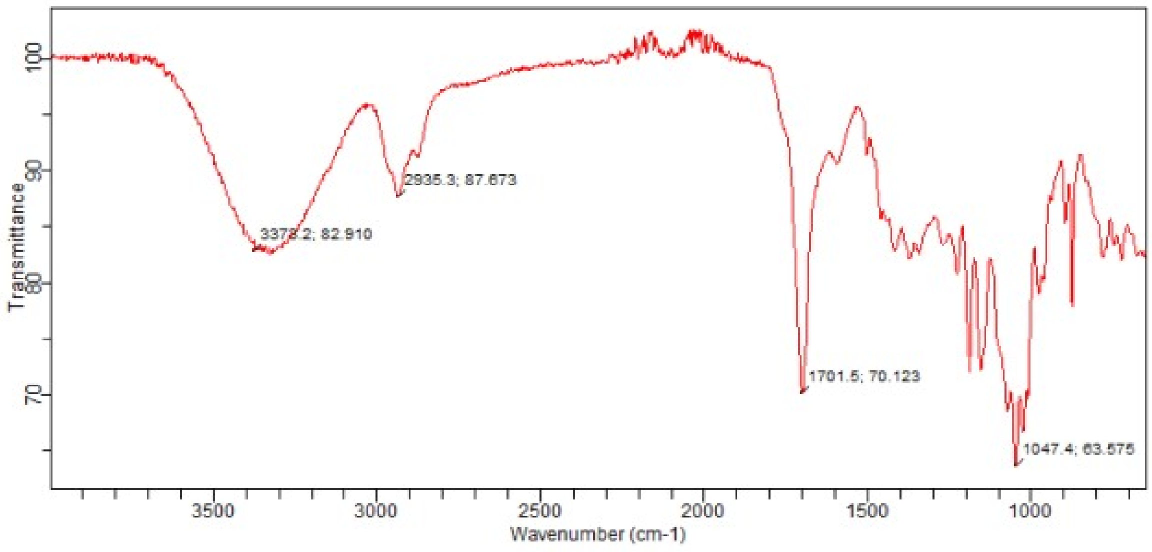

Fig. S9. HR-ESI^{-}MS [M-H]$^{-}$ spectrum of compound **2**

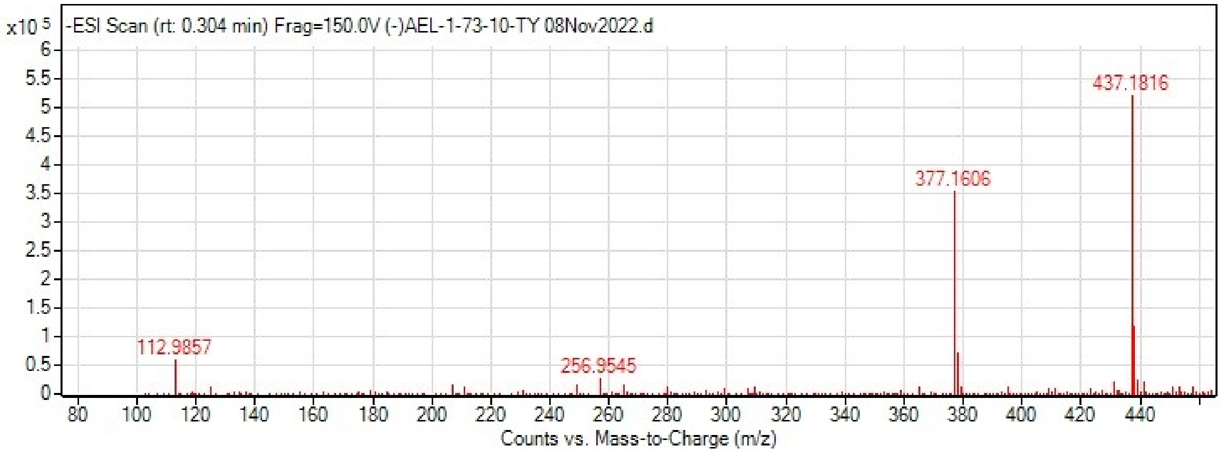

Fig. S10. ^{1}H-NMR spectrum of compound **2** (CD_3OD, 400 MHz)

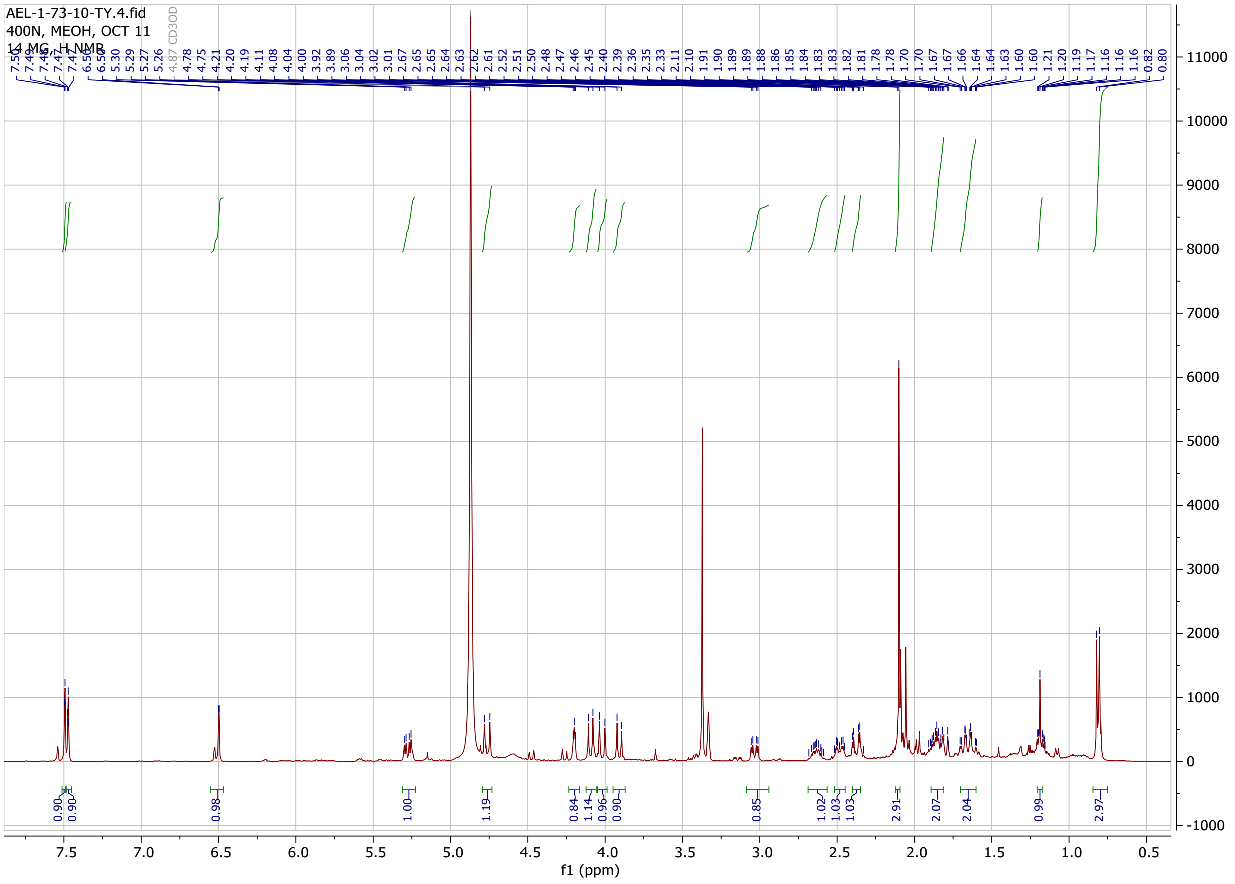

Fig. S11. DEPTQ spectrum of compound **2** (CD_3OD, 100 MHz)

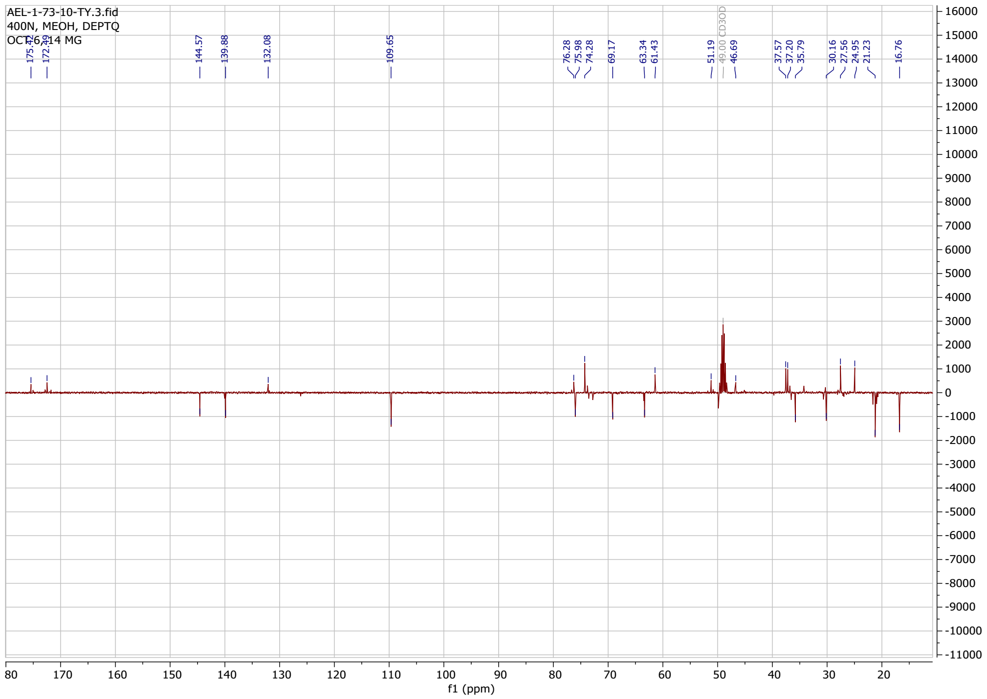

Fig. S12. ^{1}H-^{1}H COSY spectrum of compound **2** (CD_3OD, 400 MHz)

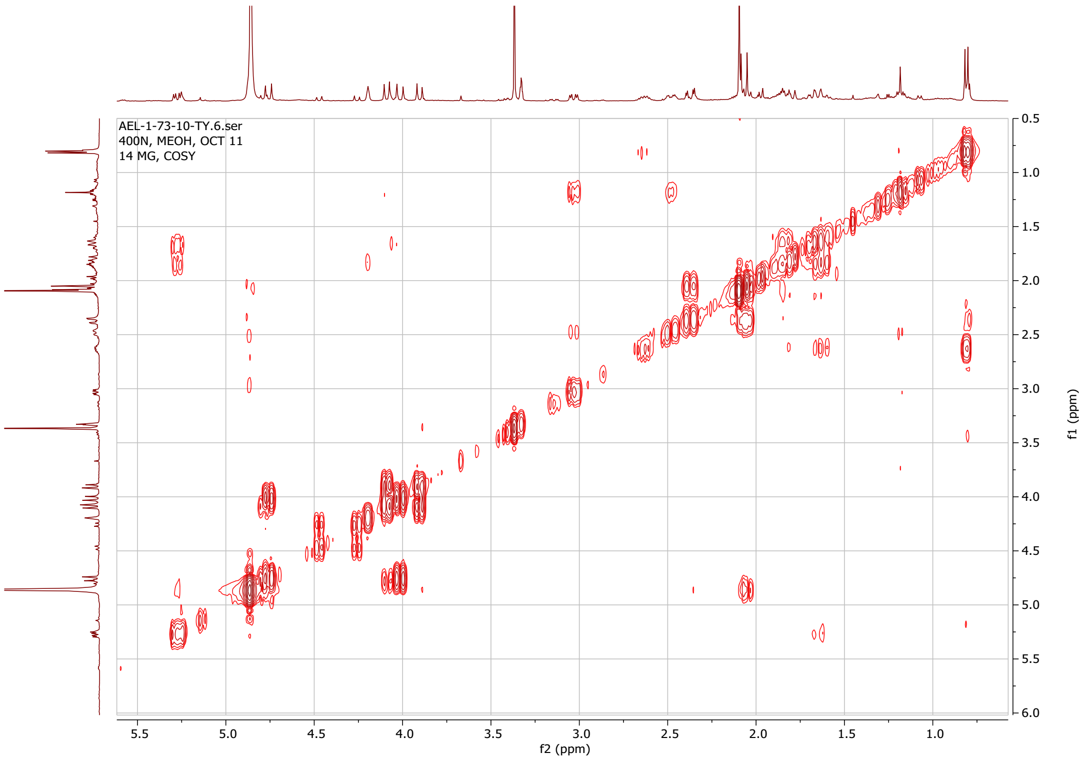

Fig. S13. HSQC spectrum of compound **2** (CD_3OD, 400 MHz)

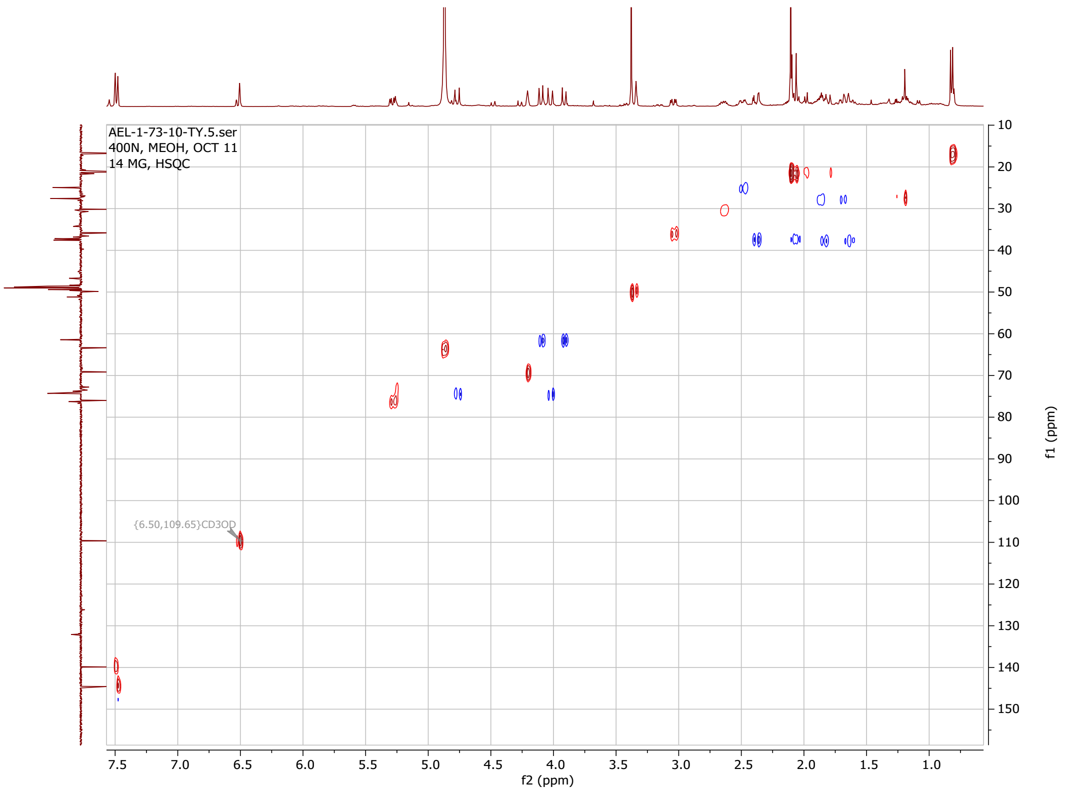

Fig. S14. HMBC spectrum of compound **2** (CD_3OD, 400 MHz)

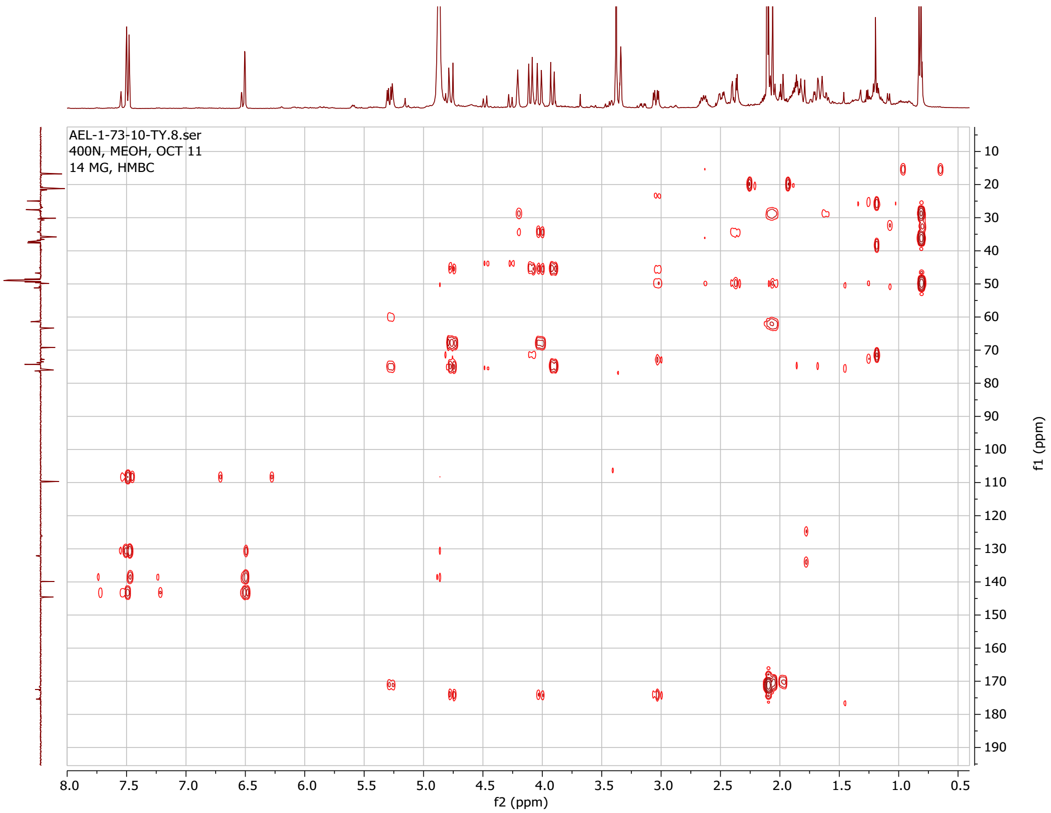

Fig. S15. NOESY spectrum of compound **2** (CD_3OD, 400 MHz)

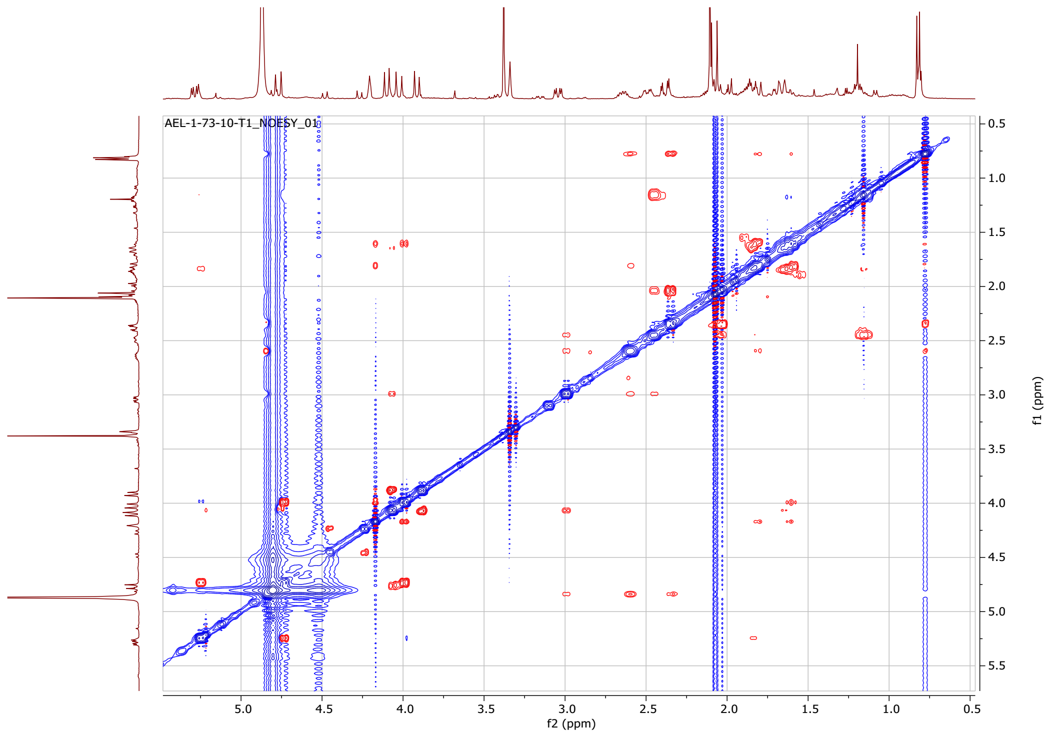

Fig. S16. IR spectrum of compound **2**

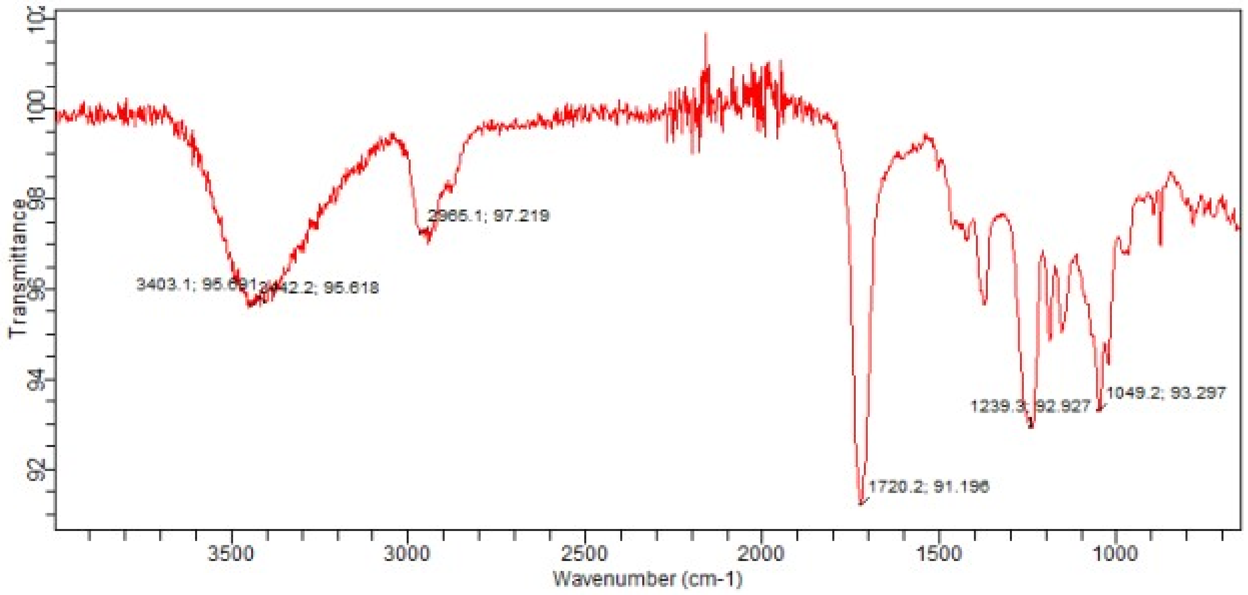

Fig. S17. HR-ESI$^-$MS [M-H]$^-$ spectrum of compound **3**

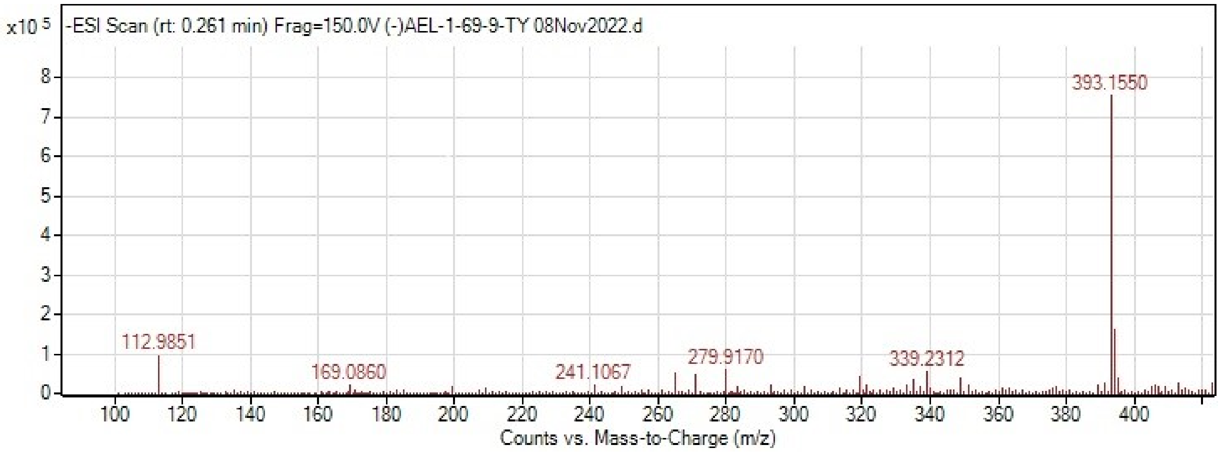

Fig. S18. ^{1}H-NMR spectrum of compound **3** (CD_3OD, 400 MHz)

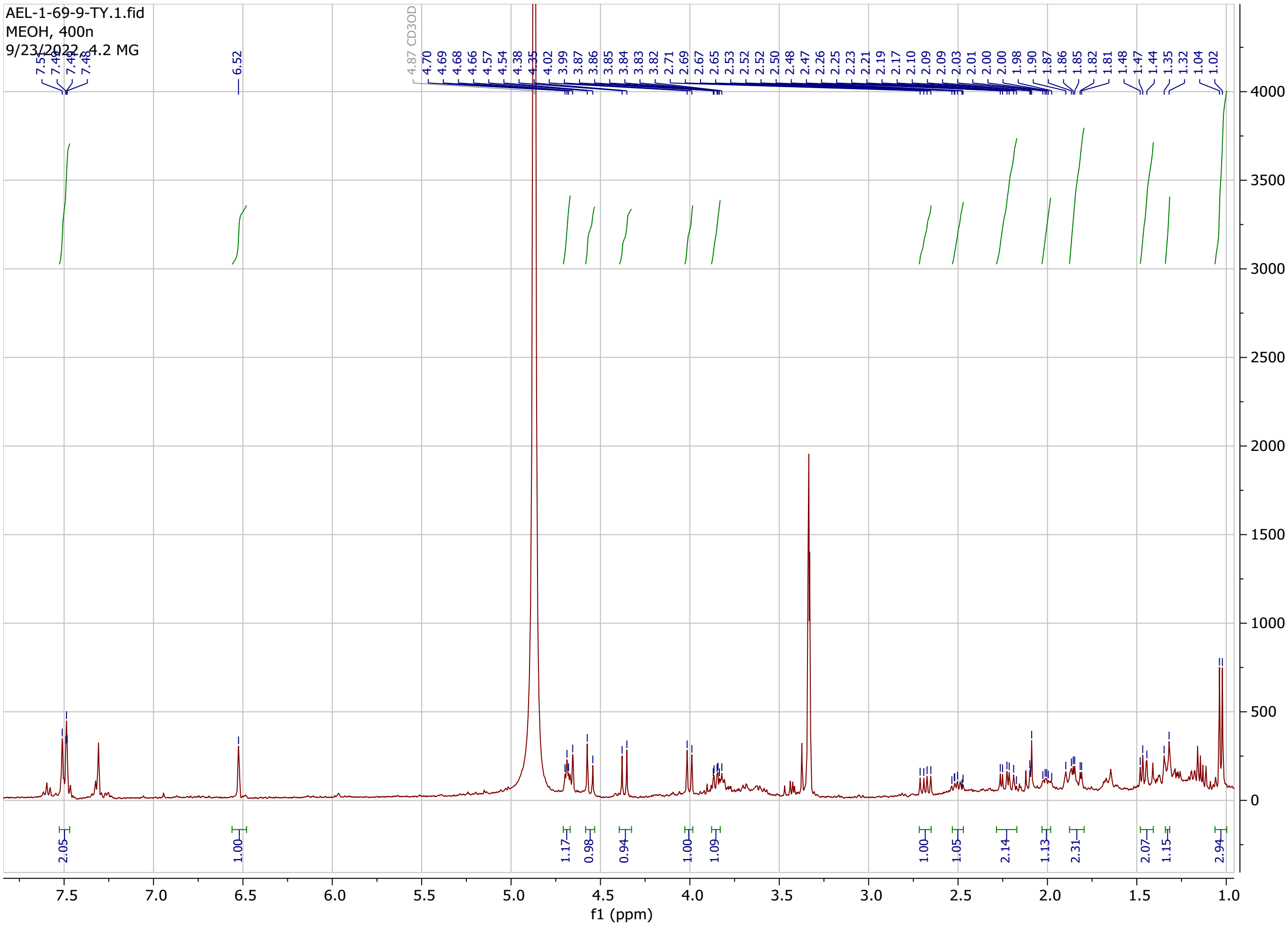

Fig. S19. DEPTQ spectrum of compound **3** (CD_3OD, 100 MHz)

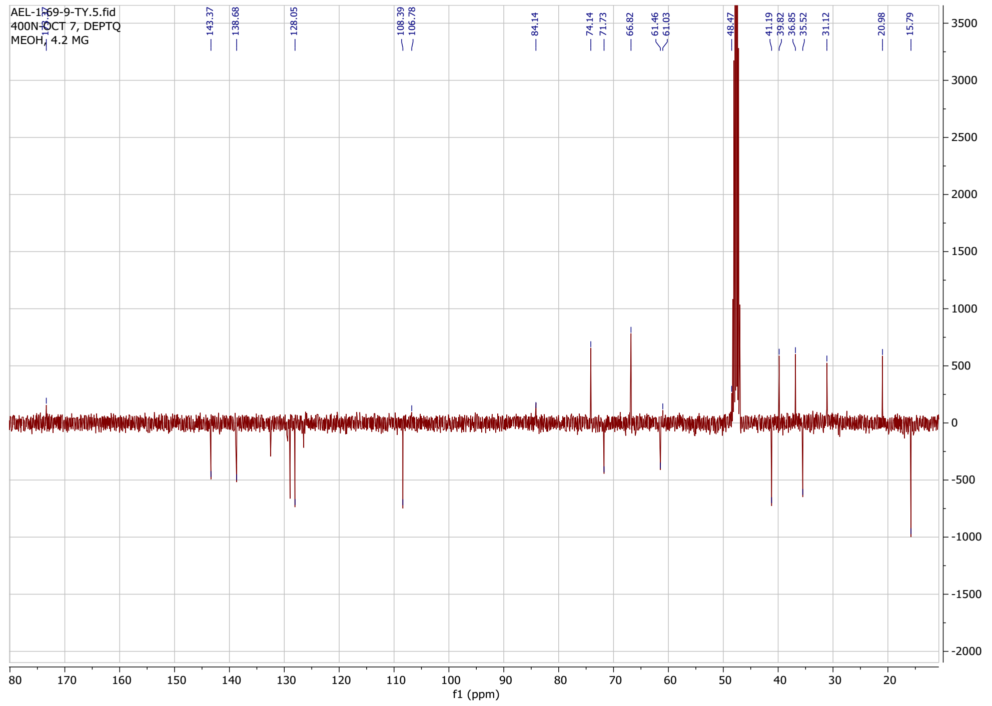

Fig. S20. ^{1}H-^{1}H COSY spectrum of compound **3** (CD_3OD, 400 MHz)

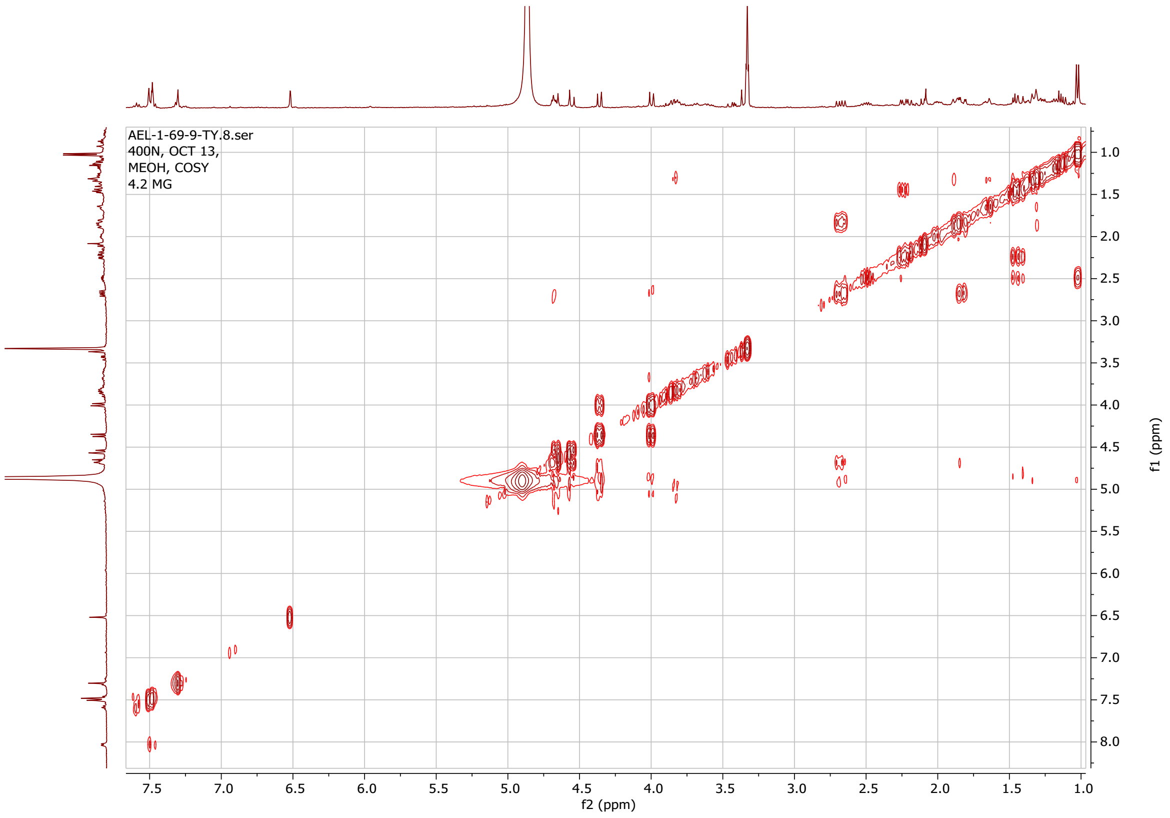

Fig. S21. HSQC spectrum of compound **3** (CD_3OD, 400 MHz)

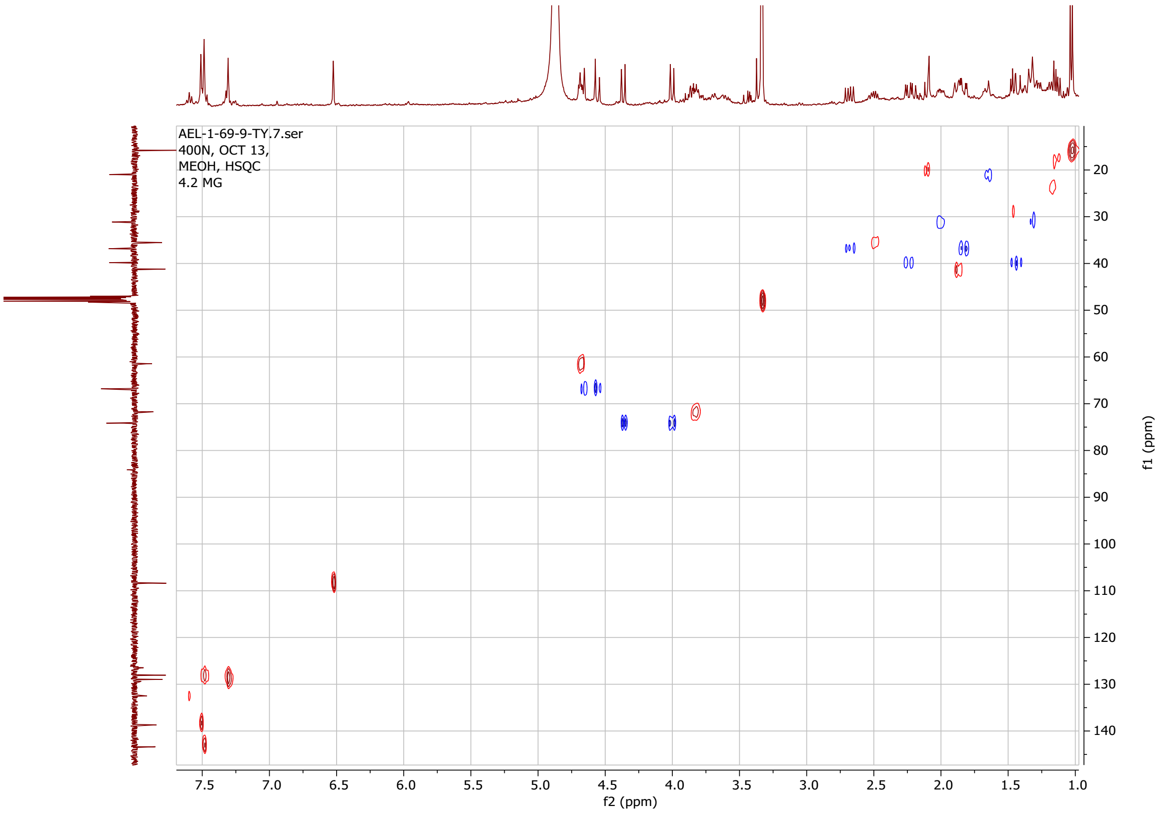

Fig. S22. HMBC spectrum of compound **3** (CD_3OD, 400 MHz)

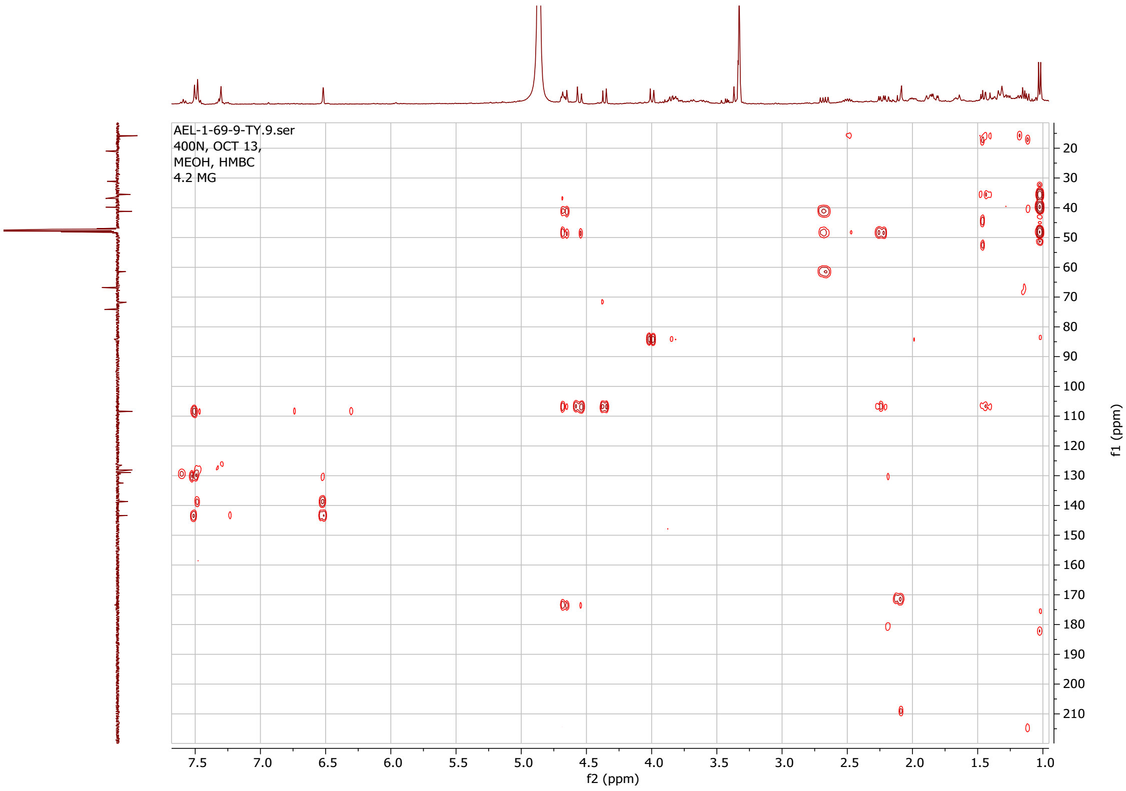

Fig. S23. HR-ESI$^-$MS [M-H]$^-$ spectrum of compound **4**

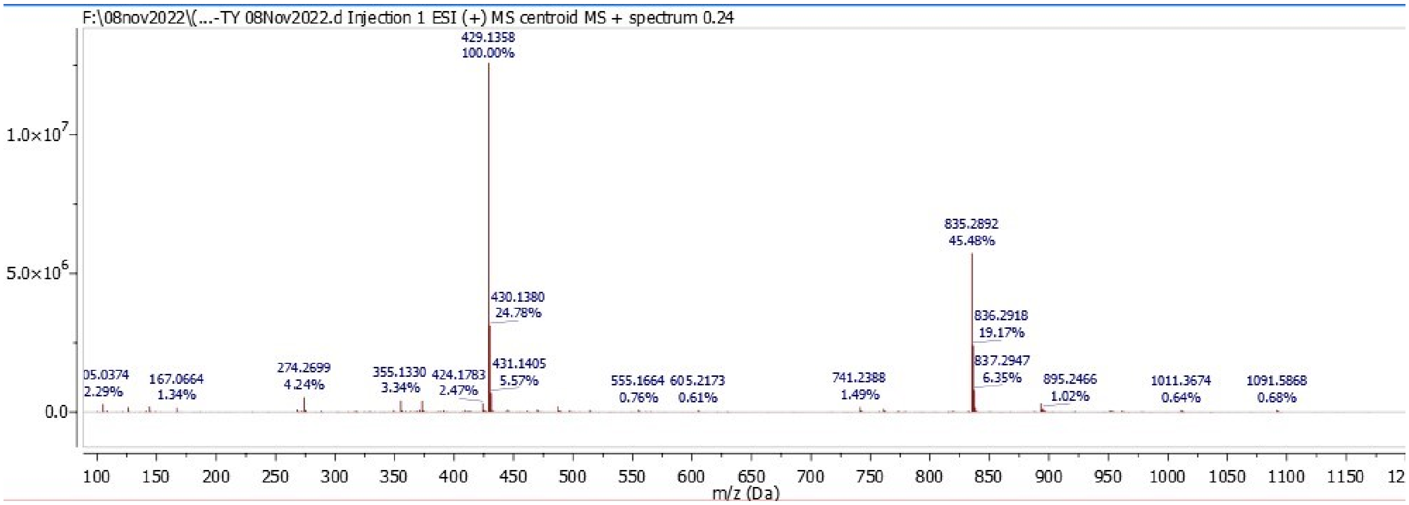

Fig. S24. ^{1}H-NMR spectrum of compound **4** (CD_3OD, 400 MHz)

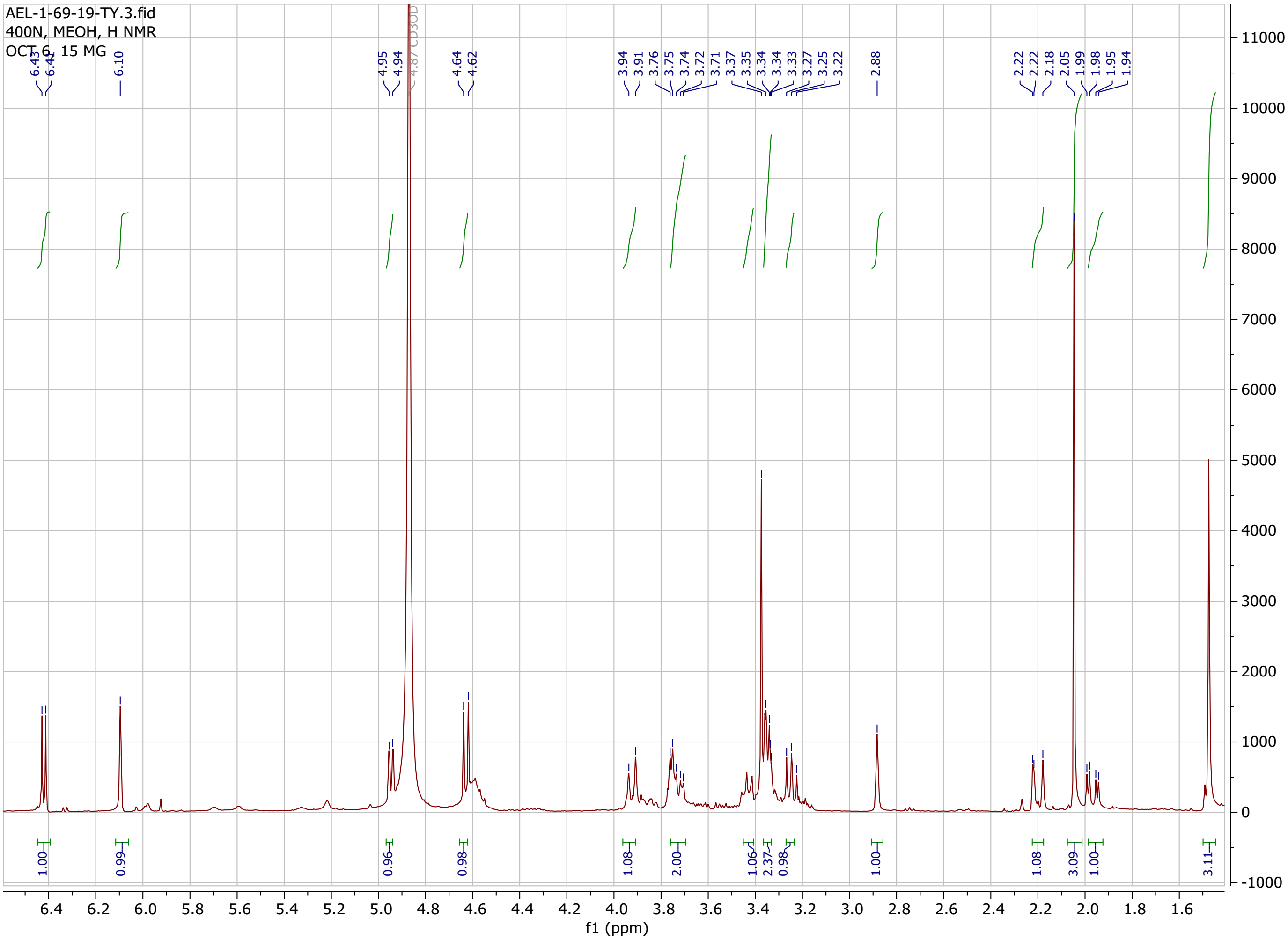

Fig. S25. DEPTQ spectrum of compound **4** (CD_3OD, 100 MHz)

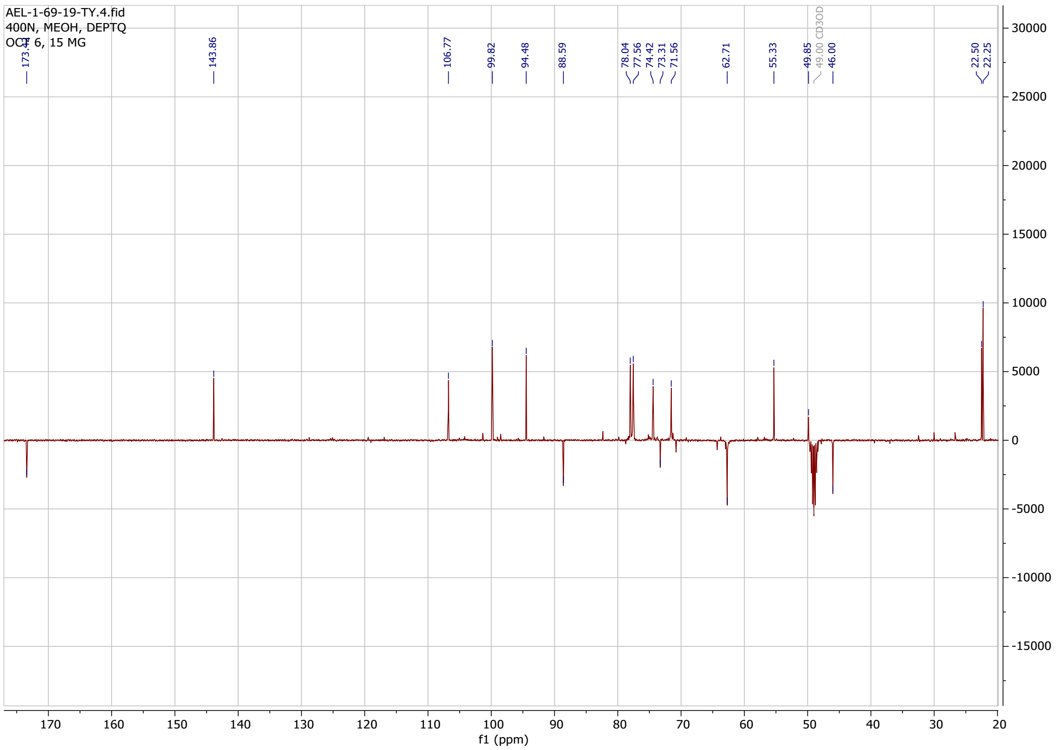

Fig. S26. HR-ESI[+]MS $[M+Na]^+$ spectrum of compound **5**

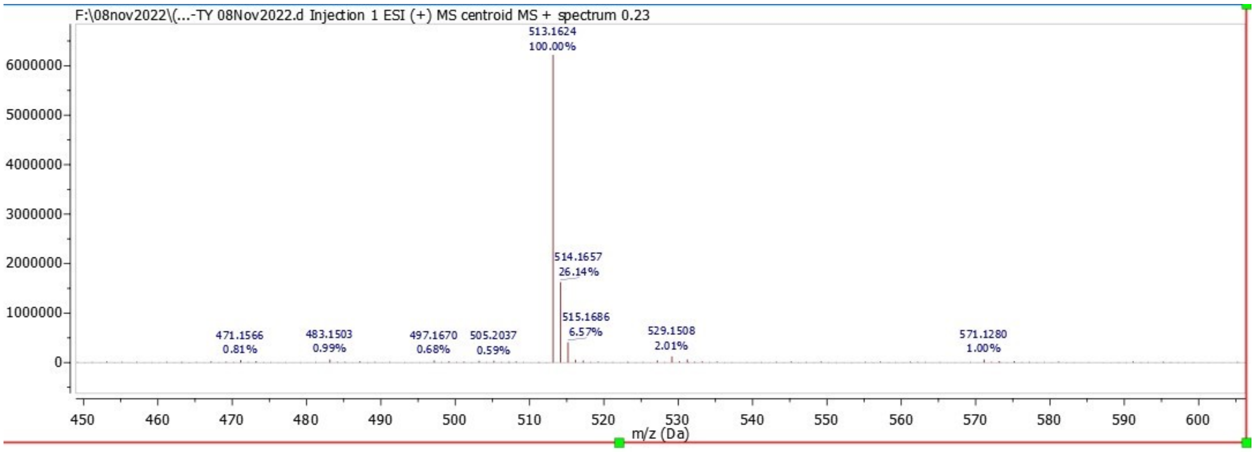

Fig. S27. ^{1}H-NMR spectrum of compound **5** (CD_3OD, 400 MHz)

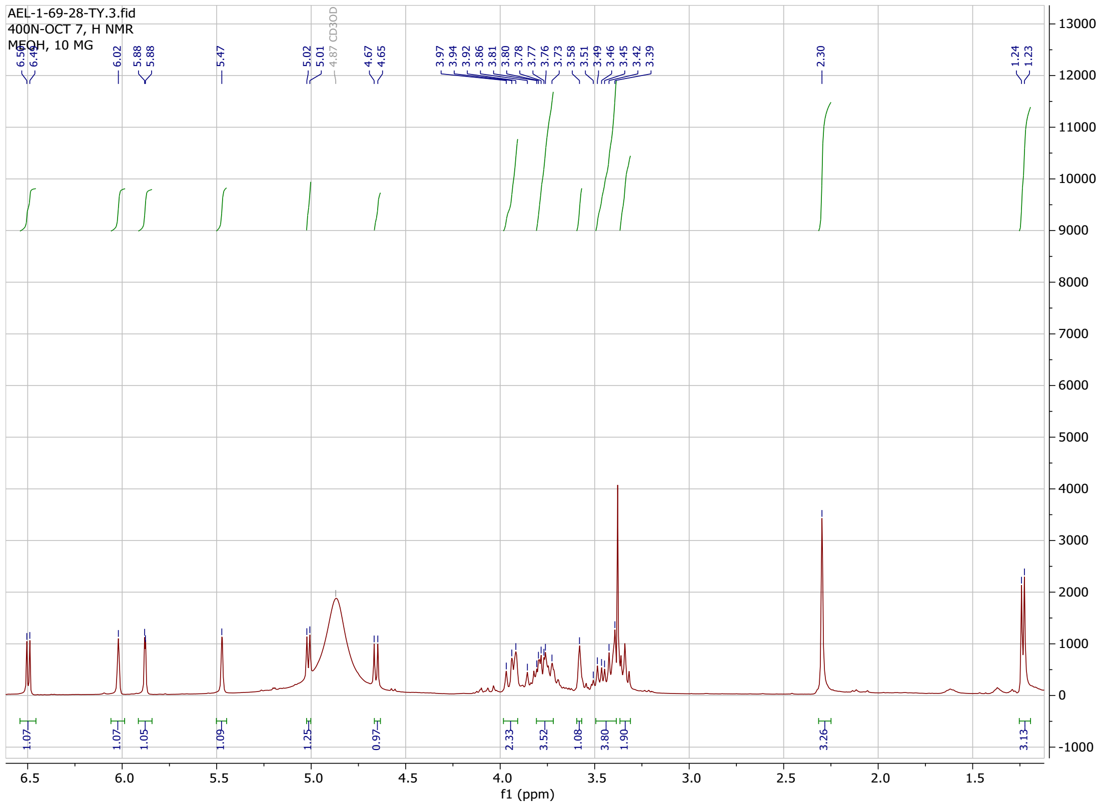

Fig. S28. DEPTQ spectrum of compound **5** (CD_3OD, 100 MHz)

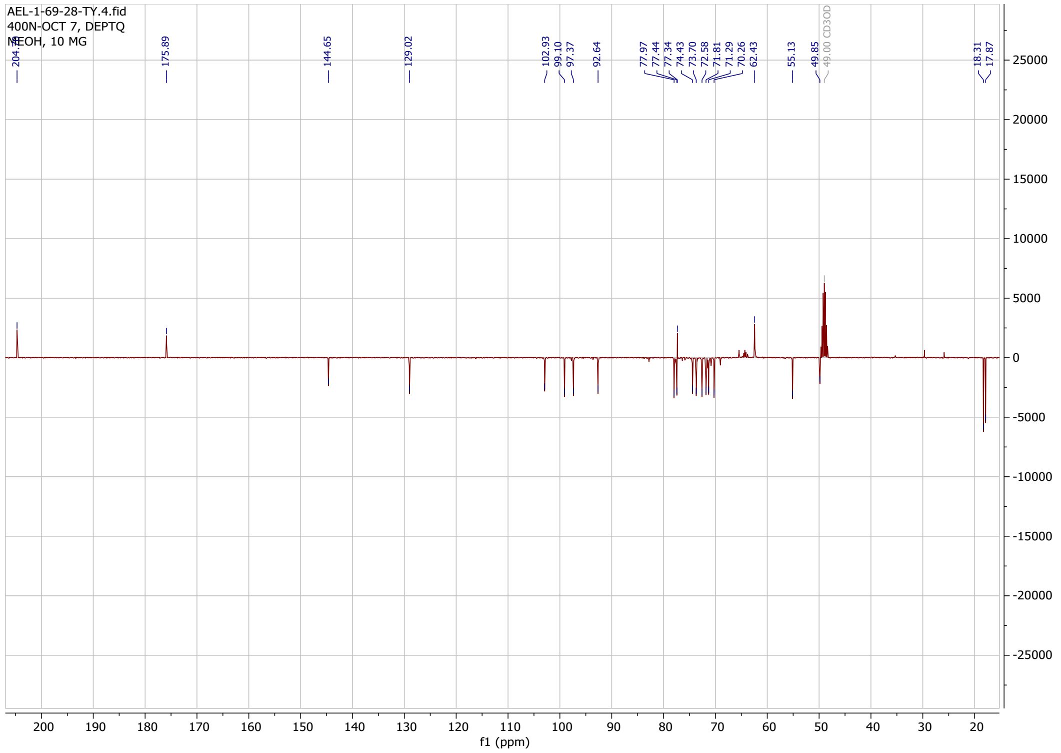

Fig. S29. ^{1}H-^{1}H COSY spectrum of compound **5** (CD_3OD, 400 MHz)

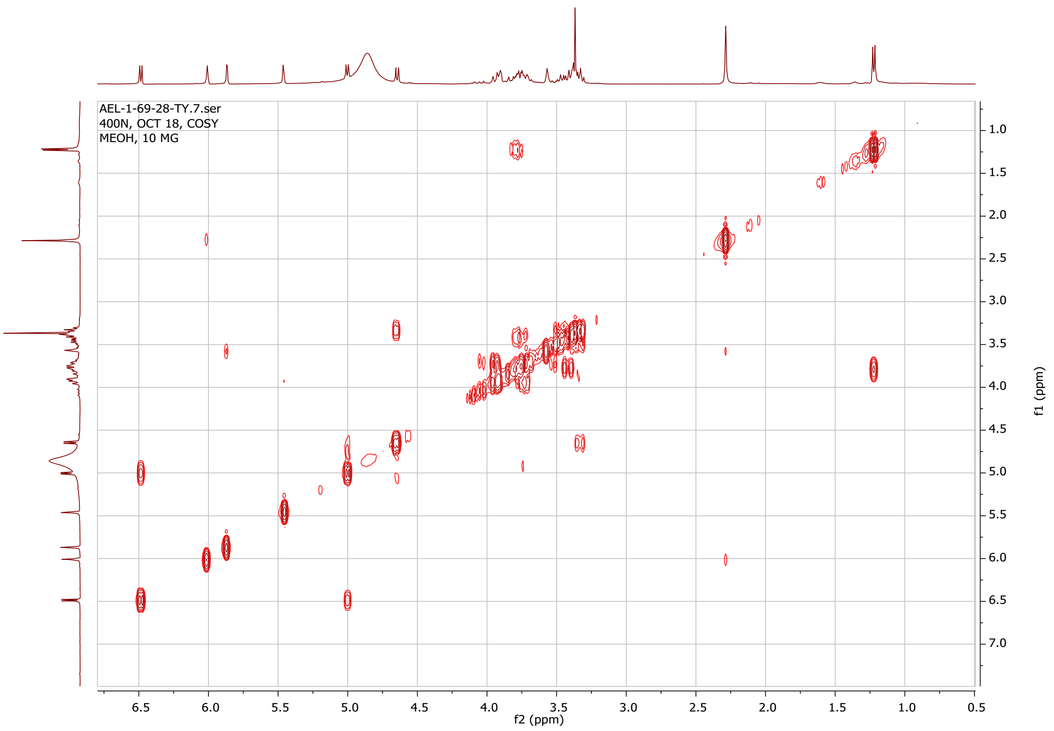

Fig. S30. HSQC spectrum of compound **5** (CD_3OD, 400 MHz)

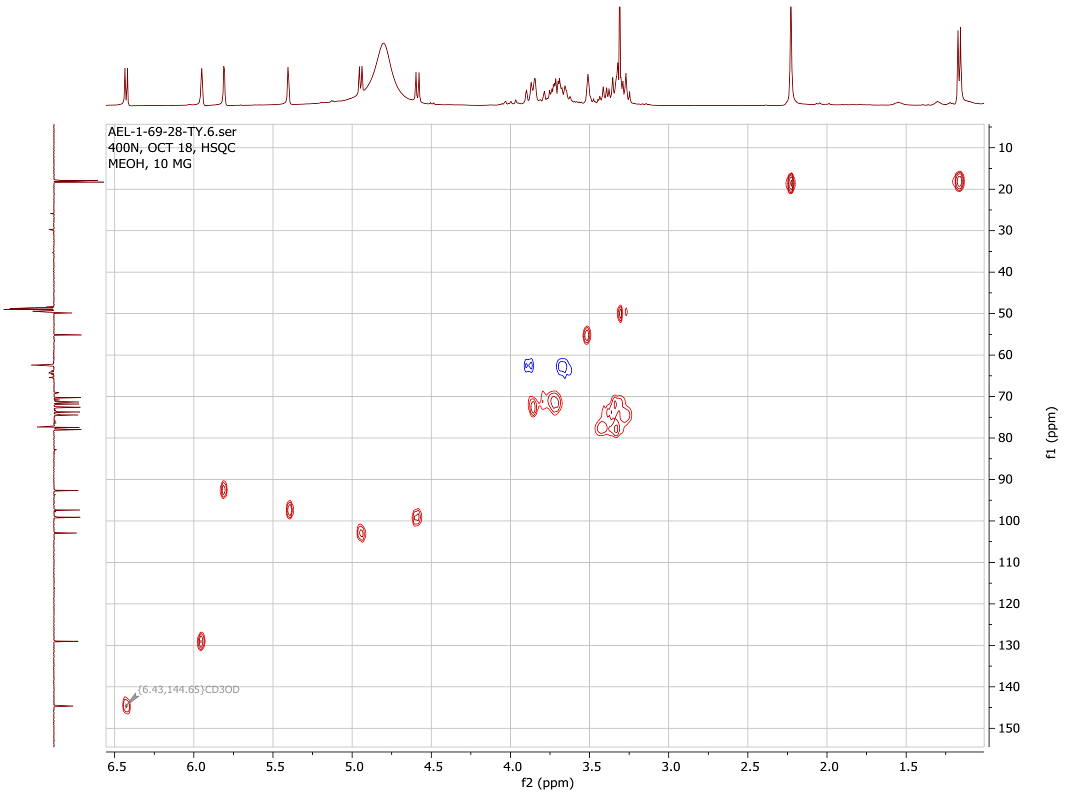

Fig. S31. HMBC spectrum of compound **5** (CD_3OD, 400 MHz)

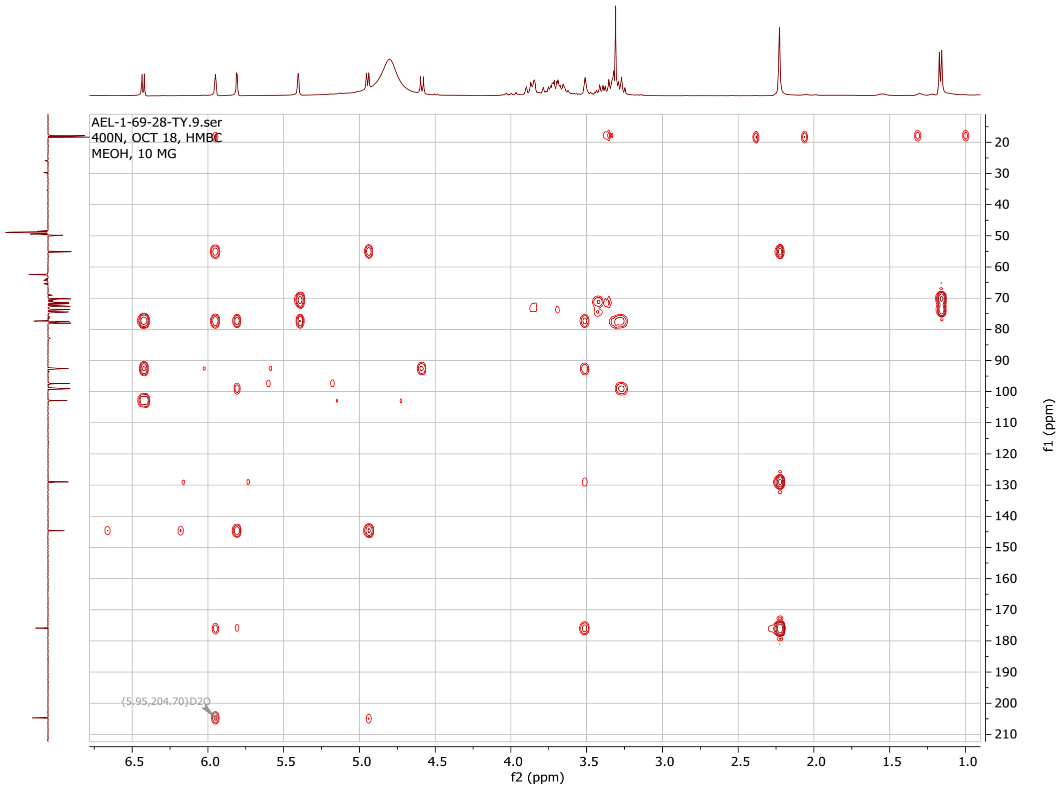

Fig. S32. Schematic isolation of compounds **1-5** from the aerial parts of *T. yemense*

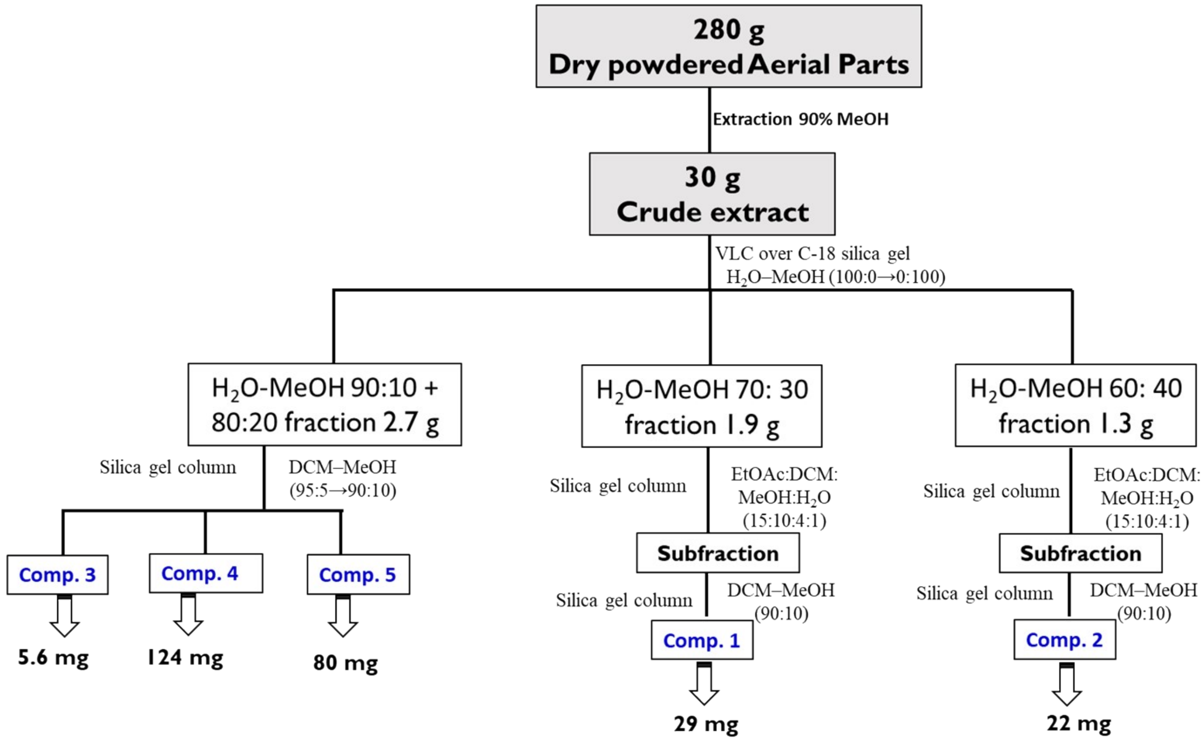

Fig. S33. HPLC chromatograms of compounds **1** and **2**

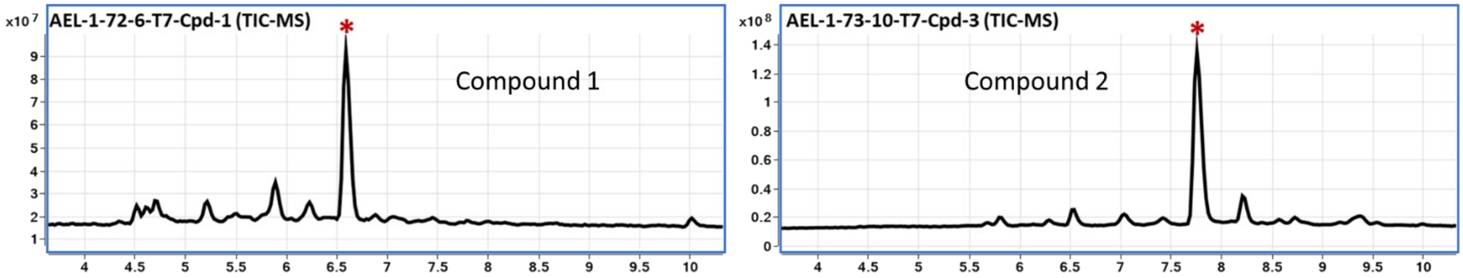

Fig. S34. Experimental ECD spectrum of compound **1**

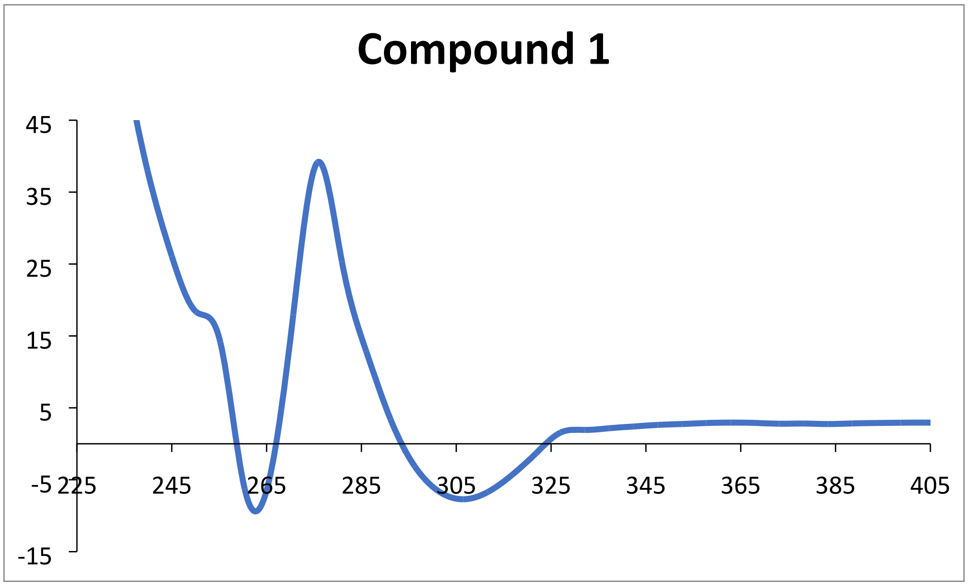

Fig. S35. Experimental ECD spectrum of compound **2**

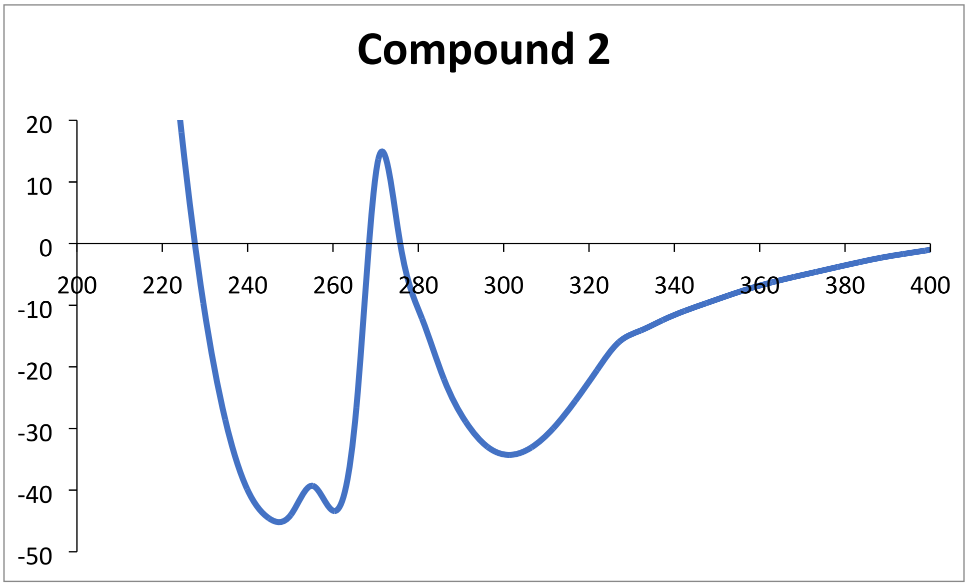

Supplement: RA-013-D3RA06083G-s001 [file RA-013-D3RA06083G-s001.pdf]
